# Functional Genomic Analysis of *Candida glabrata*-Macrophage Interaction: Role of Chromatin Remodeling in Virulence

**DOI:** 10.1371/journal.ppat.1002863

**Published:** 2012-08-16

**Authors:** Maruti Nandan Rai, Sriram Balusu, Neelima Gorityala, Lakshmi Dandu, Rupinder Kaur

**Affiliations:** Laboratory of Fungal Pathogenesis, Centre for DNA Fingerprinting and Diagnostics, Hyderabad, Andhra Pradesh, India; University of Toronto, Canada

## Abstract

Fungal septicemia is an increasingly common complication of immunocompromised patients worldwide. Candida species are the leading cause of invasive mycoses with *Candida glabrata* being the second most frequently isolated Candida species from Intensive Care Unit patients. Despite its clinical importance, very little is known about the mechanisms that *C. glabrata* employs to survive the antimicrobial and immune response of the mammalian host. Here, to decipher the interaction of *C. glabrata* with the host immune cells, we have screened a library of 18,350 *C. glabrata* Tn*7* insertion mutants for reduced survival in human THP-1 macrophages *via* signature-tagged mutagenesis approach. A total of 56 genes, belonging to diverse biological processes including chromatin organization and golgi vesicle transport, were identified which are required for survival and/or replication of *C. glabrata* in macrophages. We report for the first time that *C. glabrata* wild-type cells respond to the intracellular milieu of macrophage by modifying their chromatin structure and chromatin resistance to micrococcal nuclease digestion, altered epigenetic signature, decreased protein acetylation and increased cellular lysine deacetylase activity are the hall-marks of macrophage-internalized *C. glabrata* cells. Consistent with this, mutants defective in chromatin organization *(Cgrsc3-aΔ*, *Cgrsc3-bΔ*, *Cgrsc3-aΔbΔ*, *Cgrtt109Δ*) and DNA damage repair (*Cgrtt107Δ*, *Cgsgs1Δ*) showed attenuated virulence in the murine model of disseminated candidiasis. Further, genome-wide transcriptional profiling analysis on THP-1 macrophage-internalized yeasts revealed deregulation of energy metabolism in *Cgrsc3-aΔ* and *Cgrtt109Δ* mutants. Collectively, our findings establish chromatin remodeling as a central regulator of survival strategies which facilitates a reprogramming of cellular energy metabolism in macrophage-internalized *C. glabrata* cells and provide protection against DNA damage.

## Author Summary

Hospital-acquired fungal infections pose a colossal health and economic challenge. Candida species are the leading cause of disseminated fungal infections and rank fourth among the most common nosocomial pathogens. *C. glabrata*, an emerging opportunistic fungal pathogen, is the second most frequently isolated Candida species after *C. albicans* from Intensive Care Unit patients world-wide. Limited information is available on the unique strategies that *C. glabrata* employs to evade and replicate in host phagocytic cells since it lacks the key virulence traits of *C. albicans* including hyphal formation and secreted proteolytic activity. In the current study, we have identified a total of 56 genes, *via* a functional genomics approach, which are required for survival and/or replication of *C. glabrata* in human macrophages. Our data demonstrates an essential role for chromatin remodeling in the intracellular survival of *C. glabrata* with ingested *C. glabrata* cells displaying transcriptionally active chromatin in early-phase, compact, closed chromatin in mid-stage, and open chromatin in the late-stage of macrophage internalization. Our findings identify novel fungal virulence determinants and potentially implicate epigenetic changes in the metabolic adaptation of fungal cells to the nutrient-poor host environment and the survival against oxidative stress-induced DNA damage.

## Introduction

*Candida glabrata*, a nondimorphic, haploid budding yeast, is emerging as an important nosocomial fungal pathogen with an attributable mortality rate of ∼ 30% [1]. It normally resides as a commensal in the normal flora of human mucosal tissues but can cause infections ranging from superficial mucosal to invasive, life-threatening systemic infections in immunocompromised patients [2]. *C. glabrata* accounts for 12% of total Candida blood stream infections and is the second most common cause of candidiasis after *C. albicans* [3].

Although *C. glabrata* shares some virulence factors with *C. albicans* including phenotypic switching, bio-film formation and ability to adhere to the host tissues, it lacks key virulence traits of *C. albicans* such as hyphae formation, mating and secreted proteolytic activity [4], [5]. Despite the lack of these virulence mechanisms, *C. glabrata* is capable of establishing successful disseminated infections suggesting that it does possess a repertoire of other virulence factors. Niacin limitation responsive, chromatin-based regulation of a large family of adhesins is a unique virulence attribute of *C. glabrata* [6], [7].

Candida *spp*. are highly evolved for interaction with, and survival in, the human host. Both innate and adaptive immunity contribute towards host's resistance to candidiasis [8]. *C. albicans* is recognized primarily by two pathogen associated molecular markers, β-glucan and mannan, which constitute ∼ 90% of the cell wall dry weight [9]. The cell wall is composed of an outer layer of mannoproteins covalently linked to an inner core of β-glucan [10]. Polymorphonuclear neutrophils, macrophages and dendritic cells are natural protective barrier against systemic candidiasis and impaired phagocytic function is a major risk factor for disseminated candidiasis [11]. Dendritic cells recognize and phagocytose *C. albicans* to process them for antigen presentation and can differentiate between yeast and hyphal form to initiate the T-helper cellular immune response, which is required for long term resistance to candidiasis [12], [13].

Macrophages contribute to antifungal defense *via* phagocytosis and clearance of the fungal pathogen [14]. Nonpathogenic yeast *Saccharomyces cerevisiae* is unable to replicate in murine macrophage-like cells while *C. albicans* undergoes morphological switching and the hyphal form penetrates out of macrophages ultimately killing them [15], [16]. Contrary to this, *C. glabrata* replicates in murine macrophages and human monocyte-derived macrophages (MDMs) without inducing apoptosis or causing any significant damage to macrophages [17], [18]. Transcriptional profiling of murine macrophage-internalized *C. glabrata* cells revealed significant remodeling of carbon metabolism [17]. Recently, *C. glabrata* has been shown to modulate the phagolysosome maturation and to subvert the macrophage cytokine production [18].

Signature-tagged mutagenesis (STM) is a widely employed approach to identify virulence factors in microbial pathogens wherein multiple mutants are simultaneously screened for increased/decreased fit *via* DNA signature tags [19]. We have previously generated a Tn*7* insertion mutant library by random, *in vitro* Tn*7*-based insertional mutagenesis approach in 96-uniquely tagged wild-type strains [20]. Here, we present findings from a genomic analysis of *C. glabrata*'s interaction with human macrophages. By screening a Tn*7* insertion mutant library, representing ∼ 50% of *C. glabrata* genome, for reduced survival in a cell culture model system *via* STM approach, we have identified a set of 56 genes which are required for survival/replication in THP-1 macrophages. Further, we report for the first time that *C. glabrata* modifies its chromatin architecture in response to macrophage intracellular environment and the mutants disrupted for chromatin remodeling display survival defects in macrophages and in the murine model of systemic candidiasis. Our data suggest a link between cellular energy status and chromatin organization which is crucial for survival of *C. glabrata* in macrophages.

## Results

### *C. glabrata* survives macrophage-elicited ROS production and replicates in differentiated THP-1 cells

**Figure 1:**
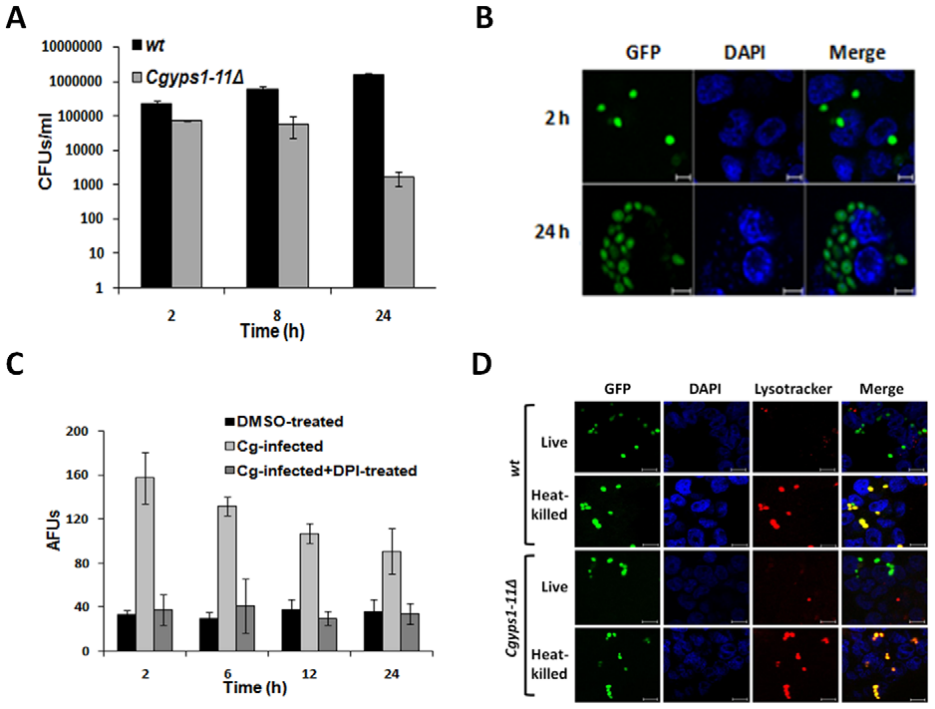
*C. glabrata* survives macrophage-elicited ROS production and replicates in differentiated THP-1 cells. (A) Colony forming unit (CFU) assay to assess the number of intracellular yeast. Data represent the mean of three to six independent analyses (± SEM). (B) Confocal images of GFP expressing yeast illustrating their replication in THP-1 macrophages. Nuclei were stained with DAPI. Scale bar = 5 µm. (C) Measurement of intracellular ROS levels by DCF (2′,7′-dichlorofluorescein) fluorescence. Diphenyleneiodonium (DPI; 10 µM) was used to inhibit ROS generation. Data are from three independent analyses ± SEM. AFU = Arbitrary fluorescence units. (D) Visualization of phagolysosome with LysoTracker Red DND99 in THP-1 macrophages which were infected with either GFP-expressing wild-type (*wt*) or mutant disrupted for aspartyl proteases (*Cgyps1–11Δ*). Scale bar = 10 µm.

To study the interaction of *C. glabrata* with human macrophages, we first established infection dynamics of *C. glabrata* cells with human monocytic cell line THP-1. Infection studies of PMA (Phorbol-12 Myristate 13-acetate)-differentiated THP-1 cells with *C. glabrata* cells at a MOI (multiplicity of infection) of 1∶10 revealed a moderate 5- to 7-fold increase in the wild-type (*wt*) colony forming units (CFUs) over a period of 24 h (Figure 1A and B) while only 1% cells remained viable for a *C. glabrata* mutant (*Cgyps1–11Δ*) which lacked eleven cell surface-associated aspartyl proteases (Figure 1A). Importantly, both *wt* and protease-defective mutant grew well in RPMI medium and were phagocytosed by THP-1 cells at a similar rate (58–62%; data not shown). Notably, replication of *C. glabrata wt* cells remained unaffected over a wide range of MOI although a few extracellular yeast cells were detected at 10∶1 MOI after 24 h of co-culturing with human macrophages (data not shown).

Confocal fluorescence microscopic analysis of *C. glabrata*-infected THP-1 macrophages revealed that two hours after co-incubation, many macrophages were infected with 1–2 yeast cells, however, 24 h post infection, the number increased to 6–10 yeast per macrophage (Figure 1B) thus corroborating the CFU-based intracellular replication of *wt C. glabrata* cells. *C. glabrata* cells were also able to replicate in mouse peritoneal macrophages wherein a 6–7 fold increase in CFUs was observed over a period of 24 h (data not shown). This ability of *C. glabrata* to replicate in activated THP-1 and mouse peritoneal macrophages is consistent with earlier findings where a 4- to 6-fold and a 3-fold increase in CFUs was observed in mouse macrophage-like cells (J774A.1) and MDMs, respectively [17]–[18]. Further, the intracellular nature of *C. glabrata* cells in THP-1 cells post 24 h infection was verified by inside/outside staining using anti-Epa1 (Epithelial adhesin 1) antibody which revealed no measurable extracellular yeast (Figure S1A).

A closer examination of *C. glabrata*'s intra-cellular behaviour *via* trypan blue staining and cell cycle analysis found 10% and 20% of *wt* cell population to be dead after 2 h and 6 h of incubation with macrophages, respectively (S1B and data not shown). However, negligible yeast cell death and a 5- to 7-fold increase in the number of internalized yeast 12 h and 24 h post infection, respectively (Figures 1A and S1B) suggested that *C. glabrata* survives an initial burst of macrophage-elicited antimicrobial response. In accord with this, *C. glabrata*-infected THP-1 and mouse peritoneal macrophages displayed a ∼ 5- and 14-fold elevation in the reactive oxygen species (ROS) levels 2 h post infection (Figure 1C and data not shown). Importantly, treatment of THP-1 cells with NADPH-oxidase inhibitor DPI (diphenyleneiodonium) reversed the ROS production to basal levels (Figure 1 C) indicating that elicitation of the oxidative burst is an active process. Consistent with above data, intracellular levels of ROS in 2 and 6 h macrophage-internalized *C. glabrata* cells were elevated by 7-fold compared to RPMI-grown cells (Figure S1C) indicating that *C. glabrata* cells encounter oxidative stress in macrophage internal milieu.

Further, labelling of *C. glabrata*-infected THP-1 cells with lysosomotropic, fluorescent dye LysoTracker Red DND99, which accumulates in acidic compartments, revealed that GFP-expressing, live *C. glabrata* cells do not co-localize with the acidic phagosomal compartment (Figure 1D). In contrast, heat-killed yeast cells exhibited co-localization with ∼ 90–95% phagosomes being lysotracker positive (Figure 1D). This suggests that live *C. glabrata* cells possess the ability to modulate the acidification of phagosome and is consistent with the findings of a recent study [18]. Surprisingly, *Cgyps1–11Δ* cells, which lost viability upon macrophage internalization, were found to be competent in preventing the maturation of phagolysosome as GFP-fluorescing live mutant cells did not co-localize with the LysoTracker stained lysosomes (Figure 1D). This implies that inhibition of phagosome acidification is one of the mechanisms that *C. glabrata* employs to survive the internal milieu of macrophages. Cytokine profiling of *C. glabrata*-infected THP-1 cells revealed an increase in the secretion of anti-inflammatory cytokine IL-4 compared to uninfected cells while no such induction was observed for other cytokines including IL-6 and IL-10 (Figure S1D and data not shown). Notably, *C. albicans* infection and LPS treatment of THP-1 cells led to the induction of IL-4 and IL-6 cytokines, respectively (Figure S1D). Together, these data suggest that activated THP-1 macrophages elicit an oxidative stress response and a modest induction of IL-4 secretion upon *C. glabrata* infection, however, *C. glabrata* manages to survive and counteract these antimicrobial responses using multiple strategies.

### Screening of the *C. glabrata* mutant library by STM approach identifies 56 genes that are required to survive/replicate in THP-1 macrophages

To identify genes involved in survival/replication of *C. glabrata* in THP-1 cells, we screened a *C. glabrata* mutant library for altered survival profiles *via* STM approach. This mutant library is composed of 18,350 mutants and was created by homologous recombination of *in vitro* generated Tn*7* insertions in the *C. glabrata* genomic clones [20]. These mutants had been assembled in a total of 192 pools wherein each pool is comprised of 96 mutants. Each of these pools carries the same set of 96 tags but within a pool each mutant contains a different tag, thus, allowing a parallel analysis of 96 mutants in each infection experiment. For a pool of tagged mutants, the ratio of hybridization in the output (macrophage-recovered yeast cells grown in YPD medium) and the input (yeast cells grown in YPD medium) pools reflected any shift in the representation of the corresponding mutant in the pool.

An MOI of 1∶10 was chosen for the mutant screen because no extracellular yeast were observed even after 48 h co-incubation with THP-1 cells (data not shown). For input, each *C. glabrata* mutant pool was grown in the YPD medium for 14 h at 37°C. For output, differentiated THP-1 cells were infected with each pool of 96-tagged mutants and intracellular yeast were recovered by lysing macrophages 24 h post infection. All the 192 mutant pools were screened and analyzed using hybridization-based STM approach. As controls for experimental reproducibility, 10 mutant pools were screened in macrophages in replicates and 15 pools were hybridized to filters in duplicates. In both cases, similar results were obtained (data not shown).

The mutants with an output/input ratio of ≥6 and ≤0.1 from the STM screen were selected as ‘up’ (increased survival) and ‘down’ (reduced survival) mutants, respectively (Tables S1 and S2). Using this cut-off value, a total of 168 (35 up and 133 down) mutants were identified which displayed altered survival profiles in differentiated THP-1 cells (Table S1). For the current study, we focussed on down mutants which displayed an output to input ratio of ≤0.1 implying at least a 10-fold underrepresentation of the unique tag in macrophage-recovered mutant cells compared to RPMI-grown mutant cells in the STM screen (Tables S1 and S2) and these, henceforth, have been referred as mutants that exhibited either≤ten-fold reduced survival or were 10-fold down in macrophages. The attenuated growth of selected mutants was validated by screening composite pools (a mixture of down mutants and tagged mutants (*wt*-like survival in macrophages) in THP-1 cells *via* hybridization-based STM manner.

Phenotypic profiling of 133 down mutants under several stressful conditions including pH and oxidative stress revealed the diverse nature of the identified mutants and overlapping sensitivities to different stresses were observed for very few mutants (Figure S2A and data not shown) thereby precluding the possibility of general sick mutants (slow-growers) coming through the screen.

Tn*7* insertion in the identified mutants was mapped, by transposon rescue and sequencing analyses, to 56 *C. glabrata* ORFs and 20 intergenic regions (http://www.genolevures.org) (Tables S1 and S2). Using the Saccharomyces Genome Database (SGD) Gene Ontology Slim Mapper (http://www.yeastgenome.org), the identified *C. glabrata* genes were functionally annotated to biological processes based upon the information available for their *S. cerevisiae* orthologs (Table S2). 12% of the identified genes were involved in chromatin organization while genes implicated in DNA repair, golgi vesicle transport, and endocytosis constituted 9%, 9% and 7%, respectively of the total identified genes (Table S2).

### Mutants defective in chromatin organization and DNA repair display reduced survival in macrophages

Of the mutants identified, we selected 10 ‘down’ mutants, which were defective for either chromatin organization or DNA replication and repair, for further analysis. The putative functions of the genes disrupted in these mutants are listed in table S2. First, we confirmed the survival defects of Tn*7* insertion mutants in THP-1 cells by single infection assays. The survival ratio of 0.4 to 0.6 for mutants defective in chromatin organization and DNA repair (Figure S2B) validated their inability to replicate properly in THP-1 cells. Liquid growth assays revealed no significant differences in the growth profiles for any mutant compared to the *wt* in YPD and RPMI medium (Figure S2C and D). *Cgdna2* mutant was exquisitely sensitive to methyl methane sulfonate (MMS), a DNA alkylating agent, while growth of *Cgchz1* mutant was impaired on plates containing MMS, hydrogen peroxide (H_2_O_2_), camptothecin (CPT), a DNA topoisomerase inhibitor and hydroxyurea (HU), a DNA replication inhibitor (Figure S2E). Surprisingly, *Cgsgs1* mutant displayed sensitivity neither to oxidative nor to replication/genotoxic stress (Figure S2E). Growth of *Cghfi1* was attenuated in the presence of H_2_O_2_ and HU. H_2_O_2_ also mildly inhibited the growth of *Cgarp7*, *Cgrtt107, Cgrtt109* and *Cgrsc3* mutants (Figure S2E). *Cgrtt107* mutant exhibited sensitivity to MMS and CPT (Figure S2E). Together, these results indicate varied levels of sensitivity of chromatin organization and DNA repair defective mutants towards genotoxic and oxidative stress causing agents.

### Chromatin is differentially modified in macrophage-internalized *C. glabrata* cells

**Figure 2:**
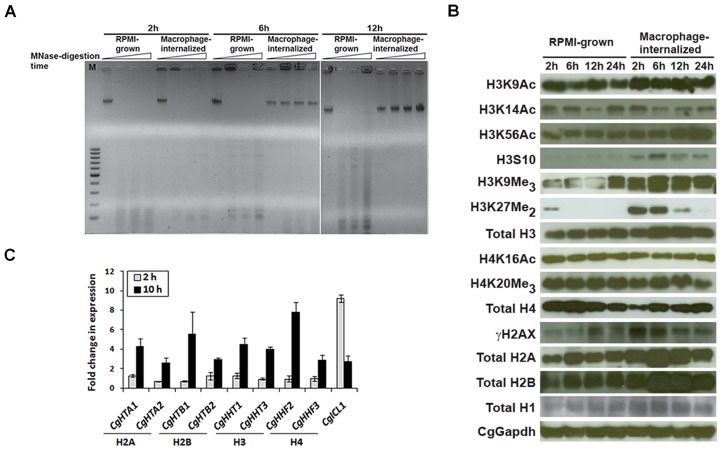
Chromatin is differentially modified in macrophage-internalized *C. glabrata* cells. (A) Chromatin from harvested cells was digested with micrococcal nuclease (MNase) at 10 units/ml for 15 min and 100 ng digested samples were resolved by agarose gel electrophoresis. (B) Immunoblot analysis on whole-cell extracts with antibodies against indicated proteins/modifications. (C) Histone mRNA levels were assessed by qRT–PCR analysis. Data (means of 3 independent experiments ± SEM) represent fold change in expression in macrophage-internalized cells compared to the RPMI-cultured cells.

As seven genes (*CgARP7*, *CgCHZ1*, *CgFPR4*, *CgHFI1*, *CgRSC3-A*, *CgRSC3-B*, *CgRTT109*), identified through the STM screen, are directly involved in maintaining chromatin architecture, we examined whether chromatin dynamics of *C. glabrata* cells is altered upon internalization by THP-1 macrophages *via* micrococcal nuclease (MNase) digestion assay. Chromatin isolated from internalized yeast post 2 h macrophage infection displayed sensitivity to MNase digestion similar to that of the RPMI-grown cells as observed by the appearance of nucleosomal bands (Figure 2A). In contrast, chromatin extracted from 6 h and 12 h macrophage-internalized yeast was highly resistant to MNase digestion (Figure 2A) and this resistance was reversed in yeast recovered 24 h post infection (Figure S3) suggesting that *C. glabrata* cells adapt to the macrophage environment by remodeling their chromatin architecture.

Biochemical post-translational modifications (PTMs) of histones H2A, H2B, H3 and H4, the protein components of the nucleosome core, contribute strongly to the structural organization of the chromatin [21]. To corroborate the altered chromatin architecture in macrophage-internalized *C. glabrata* cells, we examined both the total levels of histone proteins (H1, H2A, H2B, H3 and H4) as well as the PTMs of histones H3 and H4 in RPMI-cultured and macrophage-internalized yeast. Total levels of histones H1, H2A and H2B in macrophage-ingested yeast were elevated throughout the 24 h infection time course (Figures 2B and S4A). In contrast, H4 levels were reduced compared to the RPMI-grown cells 2, 6, and 12 h post infection (Figures 2B and S4A). Intriguingly, H3 histone levels were significantly higher 6 h and 12 h post infection (Figures 2B and S4A). This non-tandem expression of H1, H2A, H2B, and H3 with H4 was quite unexpected and the biological significance of this observation remains to be determined. Notably, elevated synthesis of histones results in chromatin condensation which may protect yeast DNA from ROS-mediated damage. Alternately, increased levels of H1, H2A, H2B and H3 may reflect the growth phase of cells as majority of histones in proliferating cells are synthesized during S-phase [22].

To investigate if changes in the histone amounts are due to their transcriptional regulation, we performed qRT-PCR analysis on 2 h and 10 h macrophage-internalized yeast. While a three- to eight-fold increase in the transcript levels of H2A, H2B, H3 and H4 coding ORFs was observed in 10 h macrophage-internalized yeast, no significant activation of the transcripts encoding histones was observed 2 h post infection (Figure 2C) suggesting that increased histone levels in 2 h macrophage-ingested yeast probably reflect stabilized proteins. The basis for the reduced levels of H4 despite the transcriptional activation of *CgHHF2* and *CgHHF3*, upon macrophage internalization is not clear and warrants further investigation.

Examination of the histone PTMs revealed that acetylation of H3 at lysine 9 and 56 was higher in macrophage internalized yeast compared to the RPMI-cultured cells, however, the acetylation was reduced by ∼ 25–50% compared to the total H3 levels 6 and 12 h post infection (Figures 2B, S4B and C). Similarly, acetylation of H3 at lysine 14 and of H4 on lysine 16 was diminished in 6 and 12 h macrophage-internalized yeast when normalized to total H3 and H4 levels, respectively (Figures 2B, S4B and C). Further, histones extracted from 2, 6 and 12 h macrophage-ingested yeast were enriched for H3K9Me_3_ and H3K27Me_2_ marks (Figures 2B, S4B, C and D). Increased phosphorylation of histone H3 at serine 10, reflective of condensed chromosomes, was also observed in internalized yeast (Figures 2B, S4B and C). Overall, these findings indicate that histone modifications that mark silent (H3K9Me_3_, H3K27Me_2_, and H4K20Me_3_) and transcriptionally active (H3K9Ac, H3K14Ac, H3K56Ac, H4K16Ac) chromatin are largely elevated and diminished in 6 and 12 h THP-1-internalized *C. glabrata* cells, respectively. Chromatin extracted from 24 h internalized yeast cells displayed predominantly an epigenetic signature (a particular pattern of DNA methylation, histone modifications and chromatin structure) of the active chromatin which is consistent with its sensitivity to MNase digestion (Figure 2A and B). Collectively, these data suggest a highly compact chromatin in 6 and 12 h macrophage-internalized *C. glabrata* cells.

### Macrophage-internalized *Cgrsc3-aΔ* and *Cgrtt109Δ* cells exhibit altered epigenetic signature

To investigate the role of chromatin remodeling in survival in THP-1 macrophages, we decided to disrupt *CgRSC3-A*, *CgRSC3-B, CgRTT107*, *CgRTT109*, and *CgSGS1* genes belonging to the GO category of either chromatin organization or DNA damage, and generated single deletion strains for these five and a double deletion strain for *CgRSC3-A* and *CgRSC3-B* genes. Our attempts to disrupt *CgARP7*, *CgCHZ1*, *CgCTI6*, *CgMRE11*, and *CgHFI1* were unsuccessful implying that these genes are probably essential in *C. glabrata*. All subsequent experiments were performed with clean deletion mutants for *CgRSC3-A*, *CgRSC3-B, CgRSC3-A* and *B, CgRTT107*, *CgRTT109*, and *CgSGS1* genes.

CgRsc3-A and CgRsc3-B are orthologs of *S. cerevisiae* Rsc3 (Table S2) which possesses sequence-specific DNA binding transcription factor activity and is a component of a 17-subunit RSC (remodel the structure of chromatin) complex [23]. The RSC ATP-dependent chromatin remodeling complex is essential for mitotic growth in *S. cerevisiae* [24]. CgRsc3-A and CgRsc3-B share an amino acid identity of 33% and 28%, respectively, with Rsc3, and 25% identity with each other. Owing to the presence of two orthologs, CgRsc3-A and CgRsc3-B in *C. glabrata* and their low levels of sequence identity with *S. cerevisiae* Rsc3, it is plausible that CgRsc3-A and CgRsc3-B may not share all functions with *S. cerevisiae* Rsc3 protein. *CgRTT109* encodes an acetyltransferase, whose counterpart in *S. cerevisiae* acetylates lysine 56 on histone H3 and functions in DNA replication and maintenance of genomic stability [25]. Orthologs of *CgRTT107* and *CgSGS1* in *S. cerevisiae* code for a BRCT (BRCA1 C Terminus) domain-containing protein and a RecQ-related nucleolar DNA helicase, respectively and are implicated in DNA double-strand break repair [26]–[27].

**Figure 3:**
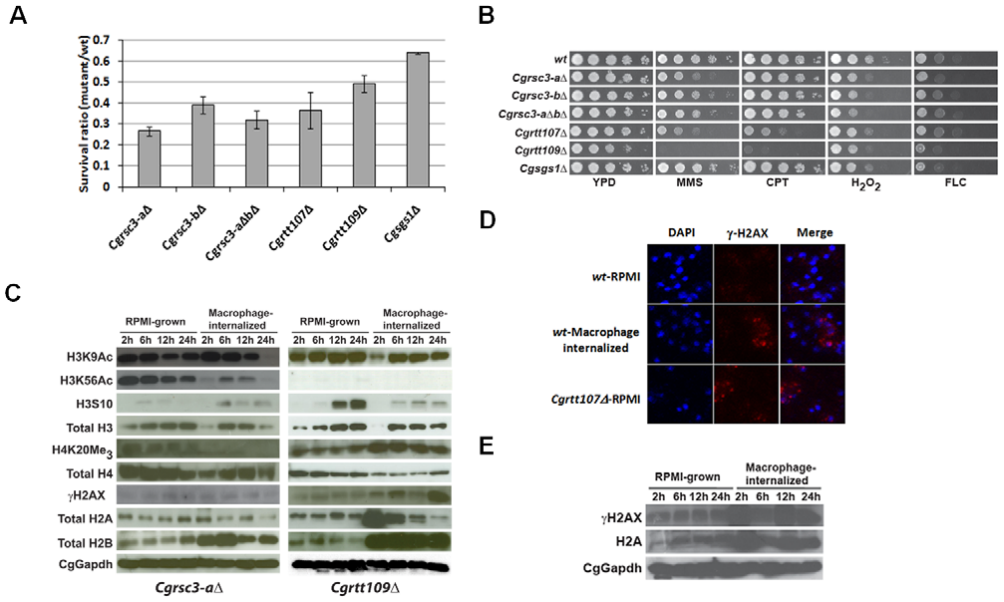
Epigenetic signature is altered in macrophage-internalized *Cgrsc3-aΔ* and *Cgrtt109Δ* mutant cells. (A) Single-strain infections of PMA-activated THP-1 cells to assess the number of intracellular yeast for mutants defective in chromatin organization. THP-1 macrophages were lysed 2 h and 24 h post infection and cell lysates were plated onto YPD medium to enumerate the viable *C. glabrata* cells. Increase in CFUs for each strain was determined by dividing the CFUs obtained at 24 h with those for 2 h. Survival ratio represent the CFU ratio of mutant/wild type after 24 h of infection. (B) Serial dilution-spotting assay to assess the growth of *C. glabrata* strains on plates containing methylmethane sulfonate (MMS; 0.03%), camptothecin (CPT; 25 µM), hydrogen peroxide (H_2_O_2_, 20 mM) and fluconazole (FLC; 16 µg/ml). (C) Immunoblot analysis on whole-cell extracts of *Cgrsc3-aΔ* and *Cgrtt109Δ* mutants using indicated antibodies. (D) Representative confocal images of γ-H2AX foci in *wt* and *Cgrtt107Δ* strains. RPMI-grown *C. glabrata* cells and *C. glabrata* cells recovered from THP-1 macrophages post 2 h infection were subjected to immunofluorescence with an anti-gamma H2A.X antibody and visualized with Alexa568-conjugated secondary antibody. Nuclei are stained with DAPI. (E) Immunoblot analysis on whole-cell extracts of *Cgrtt107Δ* mutant using indicated antibodies.

Since the STM screen was based on competitive growth assays and the mutants were selected *via* a DNA hybridization-based approach, the reduced survival/replication of *Cgrsc3-aΔ, Cgrsc3-bΔ, Cgrtt107Δ*, *Cgrtt109Δ* and *Cgsgs1Δ* mutants was examined by infecting THP-1 cells singly with either *wt* or mutant strain (Figure 3A). These single infection assays validated the replication defects of *Cgrsc3-aΔ, Cgrsc3-bΔ, Cgrtt107Δ*, *Cgrtt109Δ* and *Cgsgs1Δ* mutants in THP-1 cells with mutants displaying survival ratio of 0.3 to 0.6 (Figure 3A). Further, since both *Cgrsc3-aΔ* and *Cgrsc3-bΔ* mutants displayed growth defects in macrophages, the effect of simultaneous disruption of both genes may have been additive. However, contrary to the expectation, deletion of the two ORFs coding for CgRsc3 did not aggravate the replication defect in *Cgrsc3-aΔbΔ* mutant (Figure 3A) implying functional redundancy between CgRsc3 homologs. Importantly, all strains displayed similar growth profiles in YPD and RPMI medium (Figure S5A and B). *Cgrtt107Δ* and *Cgrtt109Δ* exhibited sensitivity to MMS, CPT and H_2_O_2_ and this susceptibility was complemented by expressing the respective ORF from the plasmid (Figures 3B and S5C). Sensitivity of *Cgrtt107Δ* and *Cgrtt109Δ* mutants to H_2_O_2_ was also confirmed by performing liquid growth assays (Figure S5D). Compared to 14% growth inhibition for *wt* cells, H_2_O_2_ treatment led to 27% and 33% growth attenuation in *Cgrtt107Δ* and *Cgrtt109Δ* mutants, respectively (Figure S5D). *Cgrtt109Δ* mutant also displayed sensitivity to fluconazole (Figure 3B). Although growth of *Cgrsc3-aΔ*, *Cgrsc3-bΔ*, *Cgrsc3-aΔbΔ* was impaired in the presence of H_2_O_2_, sensitivity to replication and DNA damage stress was not observed (Figure 3B and data not shown). Surprisingly, *Cgsgs1Δ*, unlike its *S. cerevisiae* counterpart, grew like *wt* in the presence of DNA damaging and oxidative stress causing agents (Figure 3B and data not shown) suggesting that CgSgs1 is not essential for processing of DNA double-strand breaks in *C. glabrata*.

To demonstrate that *CgRSC3-A* and *CgRTT109* are implicated in maintaining chromatin structure in *C. glabrata*, we performed MNase digestion assay. Chromatin extracted from YPD-grown logarithmic-phase *Cgrsc3-aΔ*, *Cgrsc3-aΔbΔ* and *Cgrtt109Δ* cells exhibited enhanced resistance to MNase digestion (Figure S5E) suggesting an altered chromatin architecture. Next, we checked the status of histone levels and their PTMs in RPMI-grown and macrophage-internalized *Cgrsc3-aΔ* and *Cgrtt109Δ* cells. Similar to the *wt*, increased amounts of H2A and H2B and decreased levels of H4 were observed, upon macrophage ingestion, in mutants (Figures 3C, S6A and B). Surprisingly, macrophage-internalized *Cgrsc3-aΔ* and *Cgrtt109Δ* mutant cells displayed low levels of H3 after 2 h co-incubation with macrophages (Figures 3C, S6A and B) which could be due to decreased H3 transcript levels and/or protein stability. Further, while H3 levels were elevated in 6 h macrophage-ingested *Cgrtt109Δ* cells, no appreciable increase in H3 was observed post 6 h infection in *Cgrsc3-aΔ* cells (Figures 3C, S6A and B). *Cgrtt109Δ* cells showed a low level of residual acetylation of H3 on lysine 56 (Figures 3C and S5F) implicating CgRtt109 in the acetylation of H3 at lysine 56 residue. Acetylation of H3 on lysine 9 and lysine 56 was reduced in 6 h macrophage-internalized *Cgrtt109Δ* and *Cgrsc3-aΔ* cells, respectively, however, H3 was acetylated at lysine 9 in *Cgrsc3-aΔ* cells during 6–12 h period (Figures 3C, S6A and B). Notably, unlike *wt* cells, total H3 levels in *Cgrsc3-aΔ* and *Cgrtt109Δ* cells, acetylation of H3 on lysine 9 in *Cgrtt109Δ* and acetylation of H3 at lysine 56 in *Cgrsc3-aΔ* cells were lower 24 h post infection (Figures 3C, S6A and B) suggesting diminished transcriptional activity. Phosphorylation of H3 on serine 10 was observed in 12 h and 24 h RPMI-grown *Cgrtt109Δ* cells (Figures 3C and S6B). Intriguingly, tri-methylation of H4 at lysine 20 (a repressive methyl mark), upon macrophage internalization, was significantly higher and lower in *Cgrtt109Δ* and *Cgrsc3-aΔ* cells, respectively (Figures 3C, S6A and B). The implication of this observation is not clear and warrants further investigation. Together, these results indicate an altered epigenetic response of the chromatin remodeling mutants to the macrophage environment.

To examine if *C. glabrata* cells encounter oxidative stress-induced DNA damage upon macrophage ingestion, we quantified the phosphorylation of H2AX (γ-H2AX, double-strand break-induced serine-129 phosphorylated form of H2A variant) and found it to be significantly increased in macrophage-internalized *wt* cells 2 h post infection (Figures 2B and S4A and C). A 40% increase in the appearance of γ-H2AX foci, markers of DNA damage and repair, in 2 h macrophage-internalized yeasts compared to the RPMI-grown cells (Figure 3D) corroborated the activation of DNA damage signaling upon macrophage internalization. Phosphorylation of H2AX in macrophage-internalized *Cgrsc3-aΔ* cells, when normalized to total H2A levels, was comparatively less implying a subdued response to DNA damage (Figures 3C and S6A). As a control, we also assessed DNA damage in *Cgrtt107Δ* cells and found constitutively elevated numbers of γ-H2AX foci (Figure 3D). Consistent with this, phosphorylated form of H2AX was higher in RPMI-grown and 2 h macrophage-internalized *Cgrtt107Δ* cells (Figures 3E and S6C) reflective of a constitutively active DNA damage signaling thus validating the role for CgRtt107 in the repair of damaged DNA.

Collectively, these data suggest that improper activation of DNA damage response and/or chromatin remodeling may contribute to the reduced survival of *Cgrsc3-aΔ, Cgrtt107Δ* and *Cgrtt109Δ* strains in macrophages.

### Genome-wide transcriptional profiling analysis on chromatin organization defective mutants reveals deregulation of energy metabolism

To investigate if altered chromatin structure of *Cgrsc3-aΔ* and *Cgrtt109Δ* leads to differential gene expression, we performed microarray analysis on 10 h RPMI-grown and macrophage-internalized *wt* and mutant cells. The datasets of differentially regulated genes (≥2-fold change with P≤0.05) were analysed for co-regulation by hierarchical clustering and annotated with GO term for biological process.

**Figure 4:**
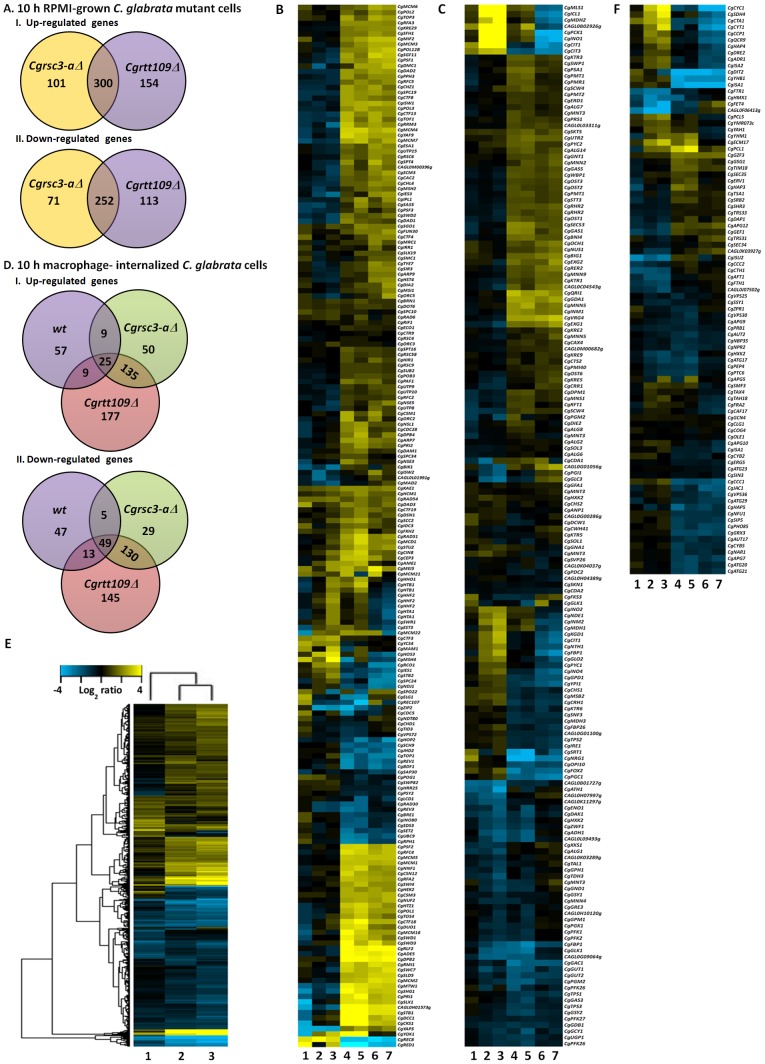
Differential gene expression pattern of *wt*, *Cgrsc3-aΔ* and *Cgrtt109Δ* cells upon macrophage ingestion. A. Venn diagram depicting the overlap between up-regulated and down-regulated genes in RPMI-grown *Cgrsc3-aΔ* and *Cgrtt109Δ* cells. B. Heat maps of expression levels of clustered and differentially expressed genes, belonging to the GO category of chromatin remodeling, in macrophage-internalized *wt* (1), *Cgrsc3-aΔ* (2) and *Cgrtt109Δ* (3) compared to the corresponding RPMI-grown cells. Lanes 4–7 represent the expression of differentially expressed genes in macrophage-internalized *Cgrsc3-aΔ* (4) and *Cgrtt109Δ* (5) and RPMI-grown *Cgrsc3-aΔ* (6) and *Cgrtt109Δ* (7) compared to the macrophage-internalized and RPMI-cultured *wt* cells, respectively. Scaled expression values are colour-coded according to the legend on the left. C. Heat maps of clustered and differentially expressed genes belonging to the GO category of cellular respiration. D. Three-way Venn diagram describing the overlap between up-regulated and down-regulated genes in macrophage-internalized *wt*, *Cgrsc3-aΔ* and *Cgrtt109Δ* cells compared to the corresponding RPMI-cultured cells. E. Dendrogram depicting hierarchical clustering of 875 differentially-expressed genes in macrophage-internalized *wt* (1), *Cgrsc3-aΔ* (2) and *Cgrtt109Δ* (3) compared to the corresponding RPMI-grown cells. F. Heat maps of clustered and differentially expressed genes belonging to the GO category of iron homeostasis.

We first compared the transcript profiles of RPMI-grown *Cgrsc3-aΔ* and *Cgrtt109Δ* cells with those of the RPMI-cultured *wt* cells and found 724 and 819 genes to be differentially regulated, respectively (Figure 4A). Intriguingly, this gene set revealed a striking overlap with 300 induced and 252 repressed genes common to both the mutants. Consistent with the role of *CgRSC3-A* and *CgRTT109* in chromatin organization, the common induced gene set included the genes involved in chromatin silencing and remodeling, RNA metabolism, ergosterol biosynthesis, DNA replication and repair (Figure 4 B and data not shown). Additionally, genes implicated in protein glycosylation were significantly represented in the induced gene dataset of *Cgrtt109Δ* mutant (Figure S7A). The repressed gene set included genes belonging to tricarboxylic acid cycle, iron-sulfur cluster assembly and mitochondrial electron transport (Figures 4C and S7B). Notably, misregulation of energy metabolism in *Cgrtt109Δ* cells is consistent with the down-regulation of the mitochondrial function-related genes observed in *rtt109^−/−^* mutant in *C. albicans* [28]. Genes implicated in DNA-dependent regulation of transcription and protein phosphorylation were uniquely down-regulated in *Cgrsc3-aΔ* and *Cgrtt109Δ* mutant, respectively. Further, expression of iron-regulated transcriptional activator CgAft2, glucose-responsive factor CgRgt1, oleate-activated factor CgOaf1 and MAPK signaling-activated transcriptional factor CgSte12 was 2- to 3-fold lower uniquely in *Cgrsc3-aΔ* mutant suggesting a positive regulatory role for *CgRSC3* in their gene expression. These data suggest that CgRsc3-a and CgRtt109-mediated chromatin organization impact transcription and exert a similar global regulatory effect on cellular physiology.

A total of 214, 432 and 683 genes were found to be differentially expressed in *wt*, *Cgrsc3-aΔ* and *Cgrtt109Δ* cells, respectively in response to macrophage internalization (Figure 4D and E). Of these, 100, 219 and 346 were induced and 114, 213 and 337 were repressed in *wt*, *Cgrsc3-aΔ* and *Cgrtt109Δ* cells, respectively. These genes were functionally annotated *via* gene ontology analysis performed with GO Slim Mapper at CGD (http://www.candidagenome.org/cgi-bin/GO/goTermMapper). The global gene expression analysis of 10 h *wt* internalized yeast revealed up-regulation of the genes involved in ammonium transport, glyoxylate cycle, β-oxidation of fatty acids, meiosis, signal transduction and proteolysis (Figures S7B, S8 and S9). The repressed genes were implicated in iron transport and homeostasis, ergosterol biosynthesis, cell wall metabolism and response to stress (Figures 4F, S8 and S9). The repression of reductive high-affinity iron assimilation in 10 h macrophage-internalized *C. glabrata* cells implies either an iron-rich internal milieu of macrophages or an anaerobic environment. Notably, iron homeostasis has previously been linked with sterol biosynthesis and oxygen availability [29].

Similar to the *wt*, macrophage-internalized *Cgrsc3-aΔ* and *Cgrtt109Δ* cells showed up-regulation of tricarboxylic acid cycle, β-oxidation of fatty acids and signal transduction, however, of total up-regulated genes, only 2% of induced genes belonged to signal transduction in *Cgrsc3-aΔ* and *Cgrtt109Δ* compared to 9% signal transduction-related up-regulated genes in *wt* (Figure S8). Further, contrary to the *wt* cells, *Cgrsc3-aΔ* and *Cgrtt109Δ* cells, upon macrophage internalization, displayed induction of the genes implicated in the generation of precursor of metabolites and energy, cellular respiration, respiratory electron transport chain and cellular amino acid metabolic process (Figures 4C, S7C, S8). Genes implicated in iron-metabolism, ergosterol biosynthesis and cell wall metabolism were down-regulated in macrophage-internalized *Cgrsc3-aΔ* and *Cgrtt109Δ* cells similar to their expression pattern in macrophage-ingested *wt* cells (Figures 4C and S7C). Interestingly, while the down-regulated gene set of *wt* cells contained 5% of translation-related genes, this gene class constituted only 1% and 2% of total repressed genes in *Cgrtt109Δ* and *Cgrsc3-aΔ*, respectively (Figure S8). Overall, microarray data indicate that transcriptional responses of *wt* and mutant cells (*Cgrsc3-aΔ* and *Cgrtt109Δ*) to macrophage internalization are largely overlapping with genes implicated in many processes including carbohydrate metabolic process, iron and ergosterol metabolism and cell wall organization displaying differential regulation (Figure S8). However, a major difference is the striking up-regulation of genes involved in generation of precursor of metabolites and energy and cellular respiration in macrophage-ingested *Cgrsc3-aΔ* and *Cgrtt109Δ* cells (Figure S8) which may lead to an imbalance between energy demand and production in mutant cells, thus, adversely affecting the intracellular survival and/or replication.

Next, we compared in parallel the mRNA profiles of macrophage-internalized *Cgrsc3-aΔ* and *Cgrtt109Δ* cells with those of the macrophage-internalized *wt* cells and found a striking overlap with a set of 355 induced and 472 repressed genes common to both mutants (Figure S9A and B). Further, up-regulation of the genes involved in DNA replication, amino acid biosynthesis, protein glycosylation and chromatin silencing and down regulation of the genes implicated in vesicle-mediated transport was observed in *Cgrsc3-aΔ* and *Cgrtt109Δ* cells in response to macrophage environment compared to the *wt* cells (Figure S7A, C and D).

Overall, ORFs either unique to *C. glabrata* or with no known function represented 15%–30% of the total differentially regulated genes among various analyzed gene sets. To validate the microarray data set, we performed qRT-PCR analysis on a set of twenty seven genes including highly up-and down-regulated genes in *wt* and metabolic and iron-responsive genes in *Cgrsc3-aΔ* and *Cgrtt109Δ* cells and observed good agreement between the microarray expression data and the transcript level measurement by qRT-PCR (Figure S9C, D and E).

Collectively, the specific up-regulation of genes implicated in cellular respiration, amino acid metabolism and chromatin silencing in macrophage-internalized *Cgrsc3-aΔ* and *Cgrtt109Δ* suggests that the mutant cells, probably due to global changes in their chromatin architecture, are unable to mount an appropriate response to restrain the cellular energy metabolism.

### Macrophage-internalized *C. glabrata* cells display elevated lysine deacetylase activity

**Figure 5:**
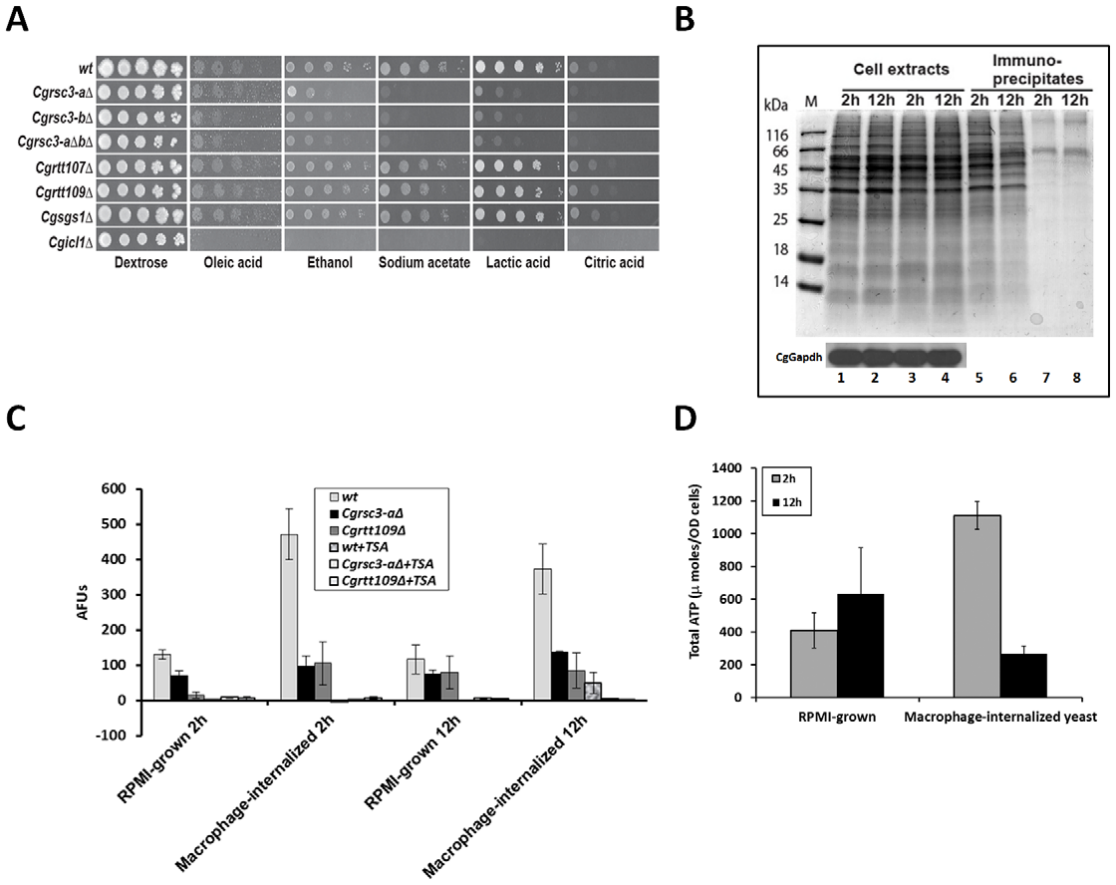
Macrophage-internalized *C. glabrata* cells display elevated lysine deacetylase activity. (A) Serial dilution-spotting assay to assess the growth of *C. glabrata* strains on plates containing indicated compounds as sole carbon sources. (B) Acetylation status of cellular proteins was checked by resolving the immunoprecipitates, pulled down with anti-acetylated lysine antibody, on SDS-PAGE. Lanes 1–2, 5–6, and 3–4, 7–8 represent RPMI-growth and macrophage internalization conditions, respectively. The supernatant fraction of the cell extracts, after immuno-precipitation with anti-acetyl lysine antibody, was probed with anti-Gapdh antibody, and similar levels of CgGapdh protein in RPMI-grown and macrophage-internalized samples were observed (bottom panel). (C) Cellular lysine deacetylase activity was measured using trifluoroacetyl-lysine as a substrate. Treatment with 10 nM trichostatin A (TSA) brought the KDAC activity to basal levels thus validating the specificity of the assay. Data represent the mean of three independent analyses (± SEM). (D) Cellular ATP was extracted from *wt* cells with trichloroacetic acid and quantified by the luciferase activity assay using the ATP bioluminescent kit. Data normalized to total viable yeast CFU counts at indicated time points is plotted and represent mean of three independent analyses (± SEM).

Reprogrammed carbon metabolism, characterized by decreased glycolysis and increased gluconeogenesis, glyoxylate cycle and fatty acid degradation is a major hallmark of macrophage-internalized fungal pathogens [15]. Thus, it is plausible that glucose limitation and/or presence of alternative carbon sources in the internal milieu of macrophages is a cue for remodeling of chromatin. To test this, we first examined the ability of the mutants defective in chromatin organization to utilize different compounds as sole carbon sources. As shown in figures 5A and S10A, *Cgrsc3-aΔ*, *Cgrsc3-bΔ*, *Cgrsc3-aΔbΔ*, *Cgchz1* and *Cghfi1* were attenuated for growth on plates containing oleic acid, ethanol, sodium acetate, citric acid and lactic acid as sole carbon source. A mutant disrupted for *CgICL1* (encodes Isocitrate Lyase, an enzyme of glyoxylate cycle) was used as a control. As expected, *Cgicl1Δ* mutant couldn't utilize any of the alternative carbon sources (Figure 5A). The inability of *Cgrsc3-aΔ*, *Cgrsc3-bΔ* and *Cgrsc3-aΔbΔ* mutants to utilize sodium acetate and lactic acid as carbon sources was also validated by liquid time course analyses (Figure S10B, C and D). Further, chromatin extracted from *wt* cells grown on sodium acetate medium displayed resistance to MNase digestion and mirrored the reduced acetylation levels of H3 on lysine 56 of macrophage-internalized cells (Figure S10E) implying a similar cellular response to macrophage environment and utilization of alternative carbon sources.

Since protein lysine acetylation is important for metabolic regulation [30], we next checked whether the overall acetylation status of yeast cellular proteins is altered in response to the macrophage environment. Significantly lower levels of lysine-acetylated proteins were observed in macrophage-internalized yeast compared to the RPMI-cultured cells (Figure 5B). In accord with this, macrophage-internalized *wt* cells displayed a 3- to 4-fold increase in the lysine deacetylase (KDAC) activity (Figure 5C) and a 1.5-fold higher intracellular ratio of NAD^+^ to NADH (data not shown) implying a low energy status. KDAC activity was also elevated when cells were grown on medium containing sodium acetate as sole carbon source (Figure S10F). Surprisingly, *Cgrsc3-aΔ* and *Cgrtt109Δ* cells did not show elevated KDAC activity upon macrophage internalization indicating an impaired epigenetic and metabolic regulation (Figure 5C). Cellular ATP measurement, to assess the energy status of macrophage-ingested cells, revealed no appreciable change and a 4-fold decrease in the total ATP levels after 2 and 12 h of co-incubation with macrophages, respectively (Figure 5D) which may be a reflection of nutrient-poor intracellular environment, low metabolic activity and/or increased ATP consumption due to activated stress responses including ATP-dependent chromatin remodeling.

### Genes implicated in chromatin organization and DNA repair are required for virulence of *C. glabrata*

**Figure 6:**
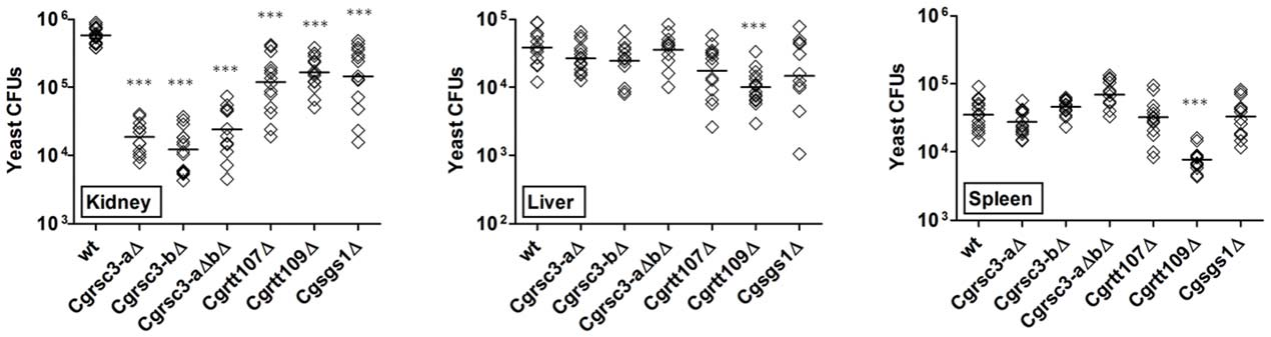
Genes involved in chromatin organization and DNA repair are required for virulence of *C. glabrata*. BALB/c mice were infected with *C. glabrata* cells intravenously and sacrificed 7 days after infection. Diamond and bar represent CFUs recovered from the target organs for individual mice and the geometric mean (n = 14–16) of the CFUs per organ, respectively. Statistical significance was determined using the Student's unpaired t-test and significant differences between mean CFUs of wild-type and mutants are labeled with a triple asterisk (P<0.0001).

To examine if chromatin remodeling is important for the virulence of *C. glabrata*, we assessed the fungal burden for *wt* and chromatin organization mutants in the murine model of systemic candidiasis. While 6×10^5^ yeast could be recovered from the kidneys of mice infected with *wt C. glabrata* cells, 25- to 50-fold lower yeast CFUs for *Cgrsc3-aΔ Cgrsc3-bΔ* and *Cgrsc3-aΔbΔ* and 3- to 5-fold lower CFUs for *Cgrtt107Δ*, *Cgrtt109Δ* and *Cgsgs1Δ* infected mice were obtained. No statistically significant differences in the fungal burden were seen in the liver and spleen of *wt*, *Cgrsc3Δ*, *Cgrtt107Δ* and *Cgsgs1Δ* infected mice (Figure 6). Interestingly, *Cgrtt109Δ* mutant showed reduced survival in all the three target organs, kidney, liver and spleen (Figure 6). Notably, similar to the survival in macrophages, an additive effect of *CgRSC3-A* and *CgRSC3-B* disruption was not observed in mice (Figure 6) suggesting a functional redundancy. Taken together, these data implicate the *CgRSC3-A, CgRSC3-B, CgRTT107*, *CgRTT109*, and *CgSGS1* genes in the survival of *C. glabrata* in the mammalian host.

## Discussion

### Identification of genes required for survival of *C. glabrata* in THP-1 macrophages

*C. glabrata* has emerged as the second most common cause of invasive candidiasis, however, our knowledge about the strategies, it employs to multiply and avoid recognition by host phagocytic cells, is very limited. To this end, we have identified a set of 56 genes, *via* STM approach, that are required for survival/replication of *C. glabrata* in human macrophages. Of these, 53 genes are novel and orthologs of *CgENA1*, *CgRTT109* and *CgSEF1* have been implicated in the virulence in *Cryptococcus neoformans* [31] and *C. albicans* [28], [32], respectively. Seven of the identified genes encode proteins which were potentially involved in chromatin organization while five of the identified gene products were implicated in DNA repair (Table S2).

As a prelude to identify strategies that *C. glabrata* might utilize to survive and replicate in macrophages, we examined, in detail, the early response of *Cgyps1–11Δ* which is unable to retain its viability in THP-1cells, to the macrophage environment. However, no appreciable differences were observed between the *wt* and *Cgyps1–11Δ* in the inhibition of phagolysosome maturation (Figure 1D), sensitivity to oxidative and hypoxic stress and utilization of alternate carbon sources (data not shown) implying defects in yet to be uncovered mechanism/s which attribute to yeast survival in THP-1 macrophages. Notably, the cytokine response of THP-1 macrophages to *Cgyps1–11Δ* infection was also similar to that upon *wt* infection (data not shown).

### Transcriptional profiling of macrophage-internalized wild-type *C. glabrata* cells

Cellular metabolism is regulated by environmental cues and the mechanisms regulating carbon and energy metabolism are tuned to sense and rapidly adapt to the varied environments. As reported previously [16]–[17], an immediate response of fungal cells to the internal milieu of macrophage is a wholesale reprogramming of carbon metabolism with elevated gluconeogenesis, β-oxidation of fatty acids and glyoxylate cycle. Our global transcript profiling analysis on 10 h macrophage-internalized *C. glabrata* cells reveals that fatty acids remained the main intracellular carbon and energy source in the macrophage milieu and yeast cells utilize the acetyl-CoA produced in fatty acid oxidation *via* glyoxylate cycle to generate energy and intermediates for synthesis of cellular building blocks (Figures 4 and S8). Further, since macrophages are postulated to be low-iron environment [33], the concerted down-regulation of genes involved in ergosterol biosynthesis, and high-affinity iron uptake and homeostasis raise the possibility of macrophage milieu being a hypoxic environment.

### Transcriptional profiling of macrophage-internalized *Cgrsc3-aΔ* and *Cgrtt109Δ* cells

In yeast, glucose acts as a source of free energy and its complete break-down to carbon dioxide and water results in the synthesis of ATP, the major energy currency molecule of the cell *via* glycolysis and aerobic respiration [34]. Reprogramming of bioenergetic pathways towards glucose metabolism is a pre-requisite for cell growth and proliferation. One of the most striking findings of our study is a significant overlap in the genes expression profiles of the *Cgrsc3-aΔ* and *Cgrtt109Δ* mutants, which displayed reduced proliferation and altered epigenetic modifications in THP-1 macrophages. Notably, *CgRSC3-A* and *CgRTT109* code for a DNA binding protein and a histone acetyltransferase, respectively, and are implicated in maintenance of the chromatin architecture.

Chromatin remodeling, the alteration of chromatin localization and structure, has long been associated with the regulation of eukaryotic gene expression [21], [35] A nucleosome which consists of 146 bp DNA and an octamer of histone proteins (histone 2A, histone 2B, histone 3, and histone 4) is the fundamental unit of chromatin [21]. Covalent post-translational modifications including acetylation, methylation, and phosphorylation of the N- and C-terminal tails of histones contribute strongly to the structural organization of chromatin [35]. Chromatin structure is pivotal for the regulation of gene expression with a compact chromatin limiting the genes' accessibility to transcription factors. Of the regulatory posttranslational modifications of histone proteins, role of methylation and acetylation in the regulation of gene expression has extensively been studied [21], [35]. Histone acetylation status is usually maintained by a dynamic equilibrium between the activity of histone acetyltransferases (HAT) and histone deacetylases (HDAC). The ‘repressed state’ of chromatin is generally equated with strict nucleosome positioning and elevated HDAC activity. Acetylation of histones at specific residues precedes the onset of transcription which is accompanied by the loosening of nucleosome positioning [21], [35].

Disruption of *CgRSC3-A* and *CgRTT109* genes led to a marked redirection of the metabolism and many energy metabolism-related genes were found to be differentially expressed in the microarray experiments. Global expression studies of RPMI-grown and macrophage-ingested *Cgrsc3-aΔ* and *Cgrtt109Δ* cells revealed down-regulation of genes involved in mitochondrial respiration under normal growth conditions and induction of genes required for the generation of precursors of metabolites and energy upon macrophage internalization implicating chromatin organization in cellular energy homeostasis. A significant overlap between transcript expression profiles of internalized mutant cells, their impaired respiratory metabolism and an inability of several chromatin organization defective mutants to utilize alternative carbon sources suggest that chromatin remodeling may act as a link between metabolic adaptation and survival in macrophages. This notion is in accord with the STM screen findings wherein 12% of the identified mutants with reduced survival in THP-1 macrophages harboured Tn*7* insertions in genes implicated in chromatin organization. Collectively, these data establish a pivotal role for the globally altered chromatin architecture in the metabolic adaptation and replication of *C. glabrata* cells in the macrophage milieu.

Of the 212 genes showing a 2-fold or greater change in the transcript abundance, upon macrophage internalization, in *C. glabrata wt* cells, we could identify the CgRsc3 binding site (Figure S9F) in 59% (153) of the genes *via in-silico* analysis indicating a central role for CgRsc3 in the regulation of gene expression. Among 153, 61 genes were up-regulated and 92 were down-regulated. Additionally, 5%, 4%, 3% and 3% of the differentially expressed genes in macrophage-internalized yeast were found to be under the control of CgMsn2, CgSte12, CgYap1 and CgSfp1 transcriptional factors, respectively (data not shown).

### Altered epigenetic signature in macrophage-ingested *C. glabrata* cells

ATP-dependent remodeling of chromatin is critical for alterations in gene expression and regulation of several physiological processes. Our data suggest that post 6 and 12 h macrophage internalization, chromatin in *C. glabrata* cells is in the ‘heterochromatic state’ with elevated repressive histone methylation and diminished euchromatic acetylation marks (Figure 2). Our data also indicate that *C. glabrata* displays a similar epigenetic response under glucose-depletion conditions where sodium acetate is the sole carbon source (Figure S10E).

**Figure 7:**
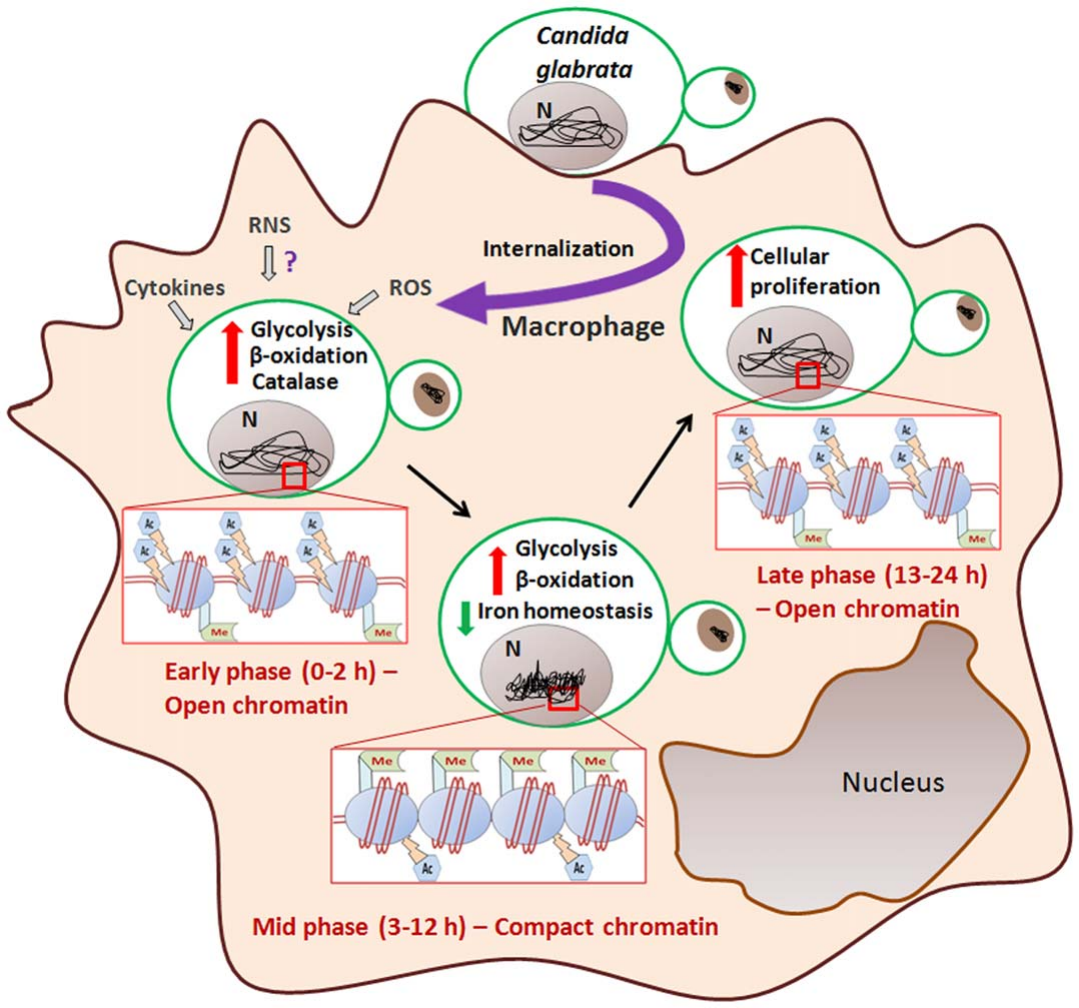
A pictorial illustration of the metabolic and epigenetic adaptation of *C. glabrata* cells to the internal milieu of THP-1 macrophages. N = nucleus.

Based on our phenotypic, microarray and biochemical analyses, we propose that *C. glabrata* cells respond to THP-1 macrophage internal milieu in three distinct phases: an Early-, a Mid- and a Late-phase (Figure 7). In the Early-phase (0–2 h), soon after phagocytosis, *C. glabrata* cells encounter oxidative stress and activate DNA repair and DNA damage signaling as characterized by elevated ROS levels and an increase in the phosphorylation of H2A at serine-129 residue and the number of γ-h2AX foci (Figures 2, S1C, and 3D). Metabolically, *C. glabrata* cells shuts down translational machinery, down regulate glycolysis and up-regulate glyoxylate and citrate cycle (data not shown) presumably mimicking a cellular response to carbon starvation. The chromatin of Early-phase *C. glabrata* cells is probably still in active conformation and exhibits sensitivity to MNase digestion (Figure 2A). During this early-phase, in response to *C. glabrata* infection, THP-1 macrophages induce production of ROS and a modest increase in the secretion of IL-4 (Figures 1C and S1D), however, they are unable to nullify the *C. glabrata*-mediated prevention of phagosome acidification.

In the Mid-phase (3–12 h), internalized *C. glabrata* cells, with a carbon metabolic profile similar to that of the early-phase, down-regulate the expression of genes implicated in iron and ergosterol biosynthesis and alter their chromatin architecture to the closed form (Figure 7). The chromatin of Mid-phase *C. glabrata* cells is resistant to MNase digestion (Figure 2A) and enriched for repressive H3 and H4 methylation marks with diminished H3 and H4 acetylation modifications at some specific residues (Figures 2B and S4). The Mid-phase may reflect the adaptive response of *C. glabrata* to macrophage environment which is characterized by presence of alternate carbon sources, high levels of ROS, and antimicrobial peptides.

The Late-phase (13–24 h) epitomizes the proliferating *C. glabrata* cells that have survived and adapted to the macrophage internal milieu (Figure 7). For the growth, cells must acquire nutrients from the host cell and activate the cell-growth related pathways. One of probable mechanisms that *C. glabrata* cells employ to proliferate in a macrophage cell is to restructure the chromatin back to open conformation which is consistent with the MNase sensitivity (Figure S3) and the histone modification patterns indicative of an active transcriptional machinery (reversal of major repressive heterochromatin marks and a modest increase in histone acetylation) of the chromatin of 24 h macrophage-ingested cells (Figure 2B).

This remodeling of chromatin during survival and/or replication in macrophages may be important for survival of macrophage-elicited ROS-induced DNA damage, rewiring of transcriptional networks to adapt to the nutrient-poor intracellular milieu, activation of stress signalling cascades and maintenance of cellular energy homeostasis and cell wall integrity. A detailed study on the effects of changes in the chromatin structure of *C. glabrata* cells on key cellular processes such as DNA replication, silencing, transcription, and stress response will yield insights into the host antimicrobial response and fungal survival strategies.

Lastly, nutritional cue-dependent reversible acetylation of major metabolic enzymes plays a pivotal role in metabolic adaptation [36]. Our data demonstrate that the total acetylation status of cellular proteins is reduced in macrophage-internalized *C. glabrata* cells which may imply a mechanism to globally regulate the activity of several key proteins at post-translational level. Although further experiments are necessary, it is plausible that deacetylation of cellular proteins including histones, in response to macrophage environment, serves three purposes; modulates the activity of metabolic enzymes, generates an acetate pool for mobilization to the generation of energy and other essential nutrients through acetyl CoA production, and modifies the chromatin to a closed, inactive form to suppress transcription, regulate cell cycle progression, and protect against DNA damage. Consistent with this, transcriptional activation of the genes, *CgACS1* and *CgACS2*, coding for acetyl-CoA synthetases was observed in response to macrophage environment. However, it remains to be investigated whether the acetate group removed by the deacetylases enters the metabolic pathways.

In conclusion, we report for the first time that chromatin remodeling contribute largely to the capability of *C. glabrata* cells to survive, function, and replicate in macrophage milieu by maintaining energy homeostasis and genes implicated in chromatin organization are required for its virulence.

## Materials and Methods

### Ethics statement

Experiments involving mice were conducted at VIMTA Labs Limited, Hyderabad in strict accordance with the guidelines of The Committee for the Purpose of Control and Supervision of Experiments on Animals (CPCSEA), Government of India. The protocol was approved by the Institutional Animal Ethics Committee (IAEC) of the Vimta Labs Ltd. (IAEC protocol approval number: PCD/OS/05). Procedures used in this protocol were designed to minimize suffering.

### Cell culture and macrophage infection assay

The human (macrophage-like) monocyte cell line THP-1 (ATCC TIB202) was maintained in RPMI-1640 medium supplemented with 10% heat-inactivated FBS and 2 mM L-glutamine at 37°C under 5% CO_2_. Briefly, 24-well plates were seeded with 1×10^6^ THP1 cells and THP-1 cells were differentiated into macrophages for 16 h in the presence of 16 nM PMA followed by 12 h recovery period. For infection assays, 50 µl of overnight grown, 0.1 OD_600_ normalized, PBS-washed *C. glabrata* cell suspensions was added to PMA-activated THP-1 cells. A range of multiplicity of infection (MOI = 1∶1, 1∶5, and 1∶10) was initially used to standardize the conditions for co-incubation and infection assay. At 2 h post-incubation, infected THP-1 cells were washed thrice with PBS to remove the non-phagocytosed yeast cells. At different times post-ingestion, PBS-washed THP-1 cells were lysed in water and the number of recovered yeast was determined by plate counts of suitable dilutions (CFU assay). Yeast grown in RPMI+serum, the culture medium for THP-1 cells, were used as *in vitro* control. The strains and plasmids used are listed in table S3. Procedures for ROS measurement and fluorescence microscopy are described in Supplemental Methods (Text S1).

### Mutant library screen

YPD-grown cultures (0.05 OD_600_) of each mutant pool (96 mutants, each carrying a unique signature tag) were either inoculated in YPD medium (input) or were used to infect differentiated THP-1 cells (1×10^6^). After 2 h incubation, non-cell-associated yeast were removed by washing THP-1 cells with PBS. At 24 h post infection, THP-1 macrophages were lysed in water and the recovered yeast cells were used to infect THP-1 cells at a MOI of 1∶10. Three rounds of macrophage infection for each pool were carried out to enrich for the desired mutants in the final population. The lysate of 3^rd^ round infection was inoculated in YPD medium for overnight (output). Cells were harvested, genomic DNA isolated from each input and output cell pellet and unique signature tags were PCR-amplified with P^32^ labelled α-dCTP using primers for the invariant region flanking each tag sequence. Labelled PCR products were denatured at 95°C for 10 min, chilled on ice and were hybridized to membrane filters, on which plasmids carrying the 96 unique tags were immobilized (detailed in Supplemental Methods (Text S1)), for 14–16 h at 42°C. The filters were washed twice and exposed to phosphorimager screen for 2–4 h. The counts for each spot were quantified using Image Quant and Fuji Multi Gauge V3.0 software. Relative percentage intensity for individual spot was calculated with respect to all the spots present on one membrane for both input and output hybridizations images.

### Micrococcal nuclease digestion

Nucleosomal-associated DNA was extracted from RPMI-grown and macrophage-internalized *C. glabrata* cells using EZ Nucleosomal DNA prep kit (ZYMO Research).

### Protein extraction and immunoblotting

Infected macrophages were lysed in ice cold water, lysate centrifugated at 4000 rpm, 4°C for 5 min and internalized yeast were collected. Yeast cells were suspended in 50 µl of protein extraction buffer (320 mM (NH_4_)_2_SO_4_, 200 mM Tris-Cl (pH 8), 20 mM EDTA (pH 8), 10 mM EGTA (pH 8), 5 mM MgCl_2_, 1 mM DTT, 10% glycerol and protease inhibitors) and disrupted using glass beads followed by centrifugation at 16000×g, 4°C for 15 min. 30 µg of total protein was resolved on a 15% SDS PAGE gel and immono-blotted with antibodies against mammalian histones/histone modifications (table S4). CgGapdh was used as a loading control. To exclude the possibility of any contribution of THP-1 proteins to the cell extracts prepared from recovered yeast, we performed three control experiments. First, probing the blots with antibodies specific for mammalian tubulin and actin yielded no signal. Second, proteinase-K treatment of lysates collected from infected macrophages, prior to the yeast pellet disruption, did not alter the epigenetic signature of *C. glabrata* cells. Lastly, probing of cell extracts, prepared from THP-1 macrophages and untreated and proteinase-K-digested *C. glabrata* cells recovered either after growth in RPMI-medium or from THP-1 macrophages, with an antibody raised against human nuclear matrix protein SATB1 gave no appreciable signal in any of the *C. glabrata* cell extracts suggesting that proteins extracted from intracellular yeasts are devoid of mammalian nuclear matrix proteins. Notably, SATB1 antibody did recognize a band of ∼ 100 kDa corresponding to the SATB1 protein of THP-1 nuclei.

### Microarray analysis

*C. glabrata* cells grown either in RPMI or harvested from THP-1 macrophages were collected, washed with DEPC treated water and were disrupted with glass beads in trizol. Total RNA was isolated using acid phenol extraction method and frozen at −80°C. The frozen RNA samples were sent to Ocimum Biosolutions Ltd., Hyderabad (http://www.ocimumbio.com). A 4×44 K GE Agilent array comprised of 10,408 probes representing 5,205 ORFs of *C. glabrata*, was used wherein average number of replicates for each probe was four to five. Feature Extraction software version 10.7.3.1. (Agilent) and Quantile normalization was used for data analysis (Text S2). Hierarchical clustering was performed using Complete Linkage method, with Euclidean Distance as distance measure. Data is the average of two hybridizations from biological replicates for each sample and raw and normalized data sets for this study are available at http://www.ncbi.nlm.nih.gov/geo/query/acc.cgi?acc=GSE38953. The protocol for qRT-PCR is described in Supplemental Methods (Text S1) and the primers used are listed in table S5.

### Mouse infection assay

Experiments involving mice were conducted at VIMTA Labs Limited, Hyderabad (www.vimta.com). Infection procedure is described in Supplemental Methods (Text S1).

### Lysine deacetylase (KDAC) activity measurement

20 µg protein samples, isolated from RPMI-grown and macrophage-internalized yeast, were taken to measure KDAC activity using HDAC Fluorimetric Assay/Drug Discovery Kit (Enzo Life Science).

#### ATP measurement

Total ATP from 1 OD yeast cells was measured using luminome tricluciferin-luciferase based assay (Adenosine 5′-Triphosphate (ATP) bioluminescent kit; Sigma).

## Supporting Information

Figure S1 ***C. glabrata*** **cells are killed by activated THP1-cells.** (**A**) Inside/Outside staining to validate intracellular replication of *C. glabrata* wild-type cells. After 24 h of infection, GFP-expressing *C. glabrata* cells were labeled with anti-Epa1 (Epithelial adhesin 1) antibody and visualized using Alexa Fluor 568-conjugated secondary antibody. Owing to inaccessibility to primary and secondary antibody, intracellular yeast fluoresced green. No doubly-labelled (red and green) extracellular yeasts were observed. The bottom panel serves as a control and confirms the reactivity of anti-Epa1 antibody in RPMI-grown *C. glabrata* wild-type cells. Epa1 being a cell surface protein, a clear distinct ring of surface-localized Epa1 was observed. (**B**) Trypan blue exclusion assay to assess the viability of RPMI-grown and macrophage-internalized *C. glabrata* cells in PMA-activated THP-1 cells. Cells were collected at indicated time points and stained with 0.4% trypan blue for 10 min. A minimum of total 300 cells (stained (dead) and unstained (viable)) were counted microscopically for data point. Cell viability data were plotted as the percentage of trypan blue positive cells and represent the mean of three independent analyses (± SEM). (**C**) Measurement of ROS levels in macrophage-internalized yeast at indicated time points post infection using the DCF (2′,7′-dichlorofluorescein) fluorescence assay. Data are from three independent analyses ± SEM. AFUs = Arbitrary fluorescence units. (**D**) *C. glabrata* elicits interleukin-4 (IL-4) production in PMA-activated THP-1 cells. PMA-activated THP1 cells infected with yeast cells at a MOI of 1∶10. Two hours post infection, cells were washed with PBS thrice and incubated in fresh RPMI medium at 37°C. As a control, THP-1 cells were treated with 1 µg/ml LPS for 24 h. After 24 h, supernatants were collected, centrifuged at 3000 rpm for 5 min to remove any particulate matter if any. Expression of indicated cytokines was enumerated using BD OptEA ELISA kit as per the supplier's instructions. To calculate the relative cytokine levels, readings were normalized to the supernatant of PMA-treated, uninfected THP-1 cells. Data represent the mean of three independent infection experiments (± SEM).
(TIF)

Figure S2 **Mutants disrupted for chromatin organization display reduced survival in macrophages and varied sensitivity to genotoxic stress.** (**A**) Heat map depicting the growth of mutants, identified through the STM screen, in the presence of diverse stresses. Since several mutants with multiple Tn*7* insertions in the same gene and twenty mutants harboring Tn*7* insertions in intergenic regions were identified in the STM screen, all these mutants were not subjected to further analysis. Instead, a total of 56 mutants carrying Tn*7* insertions in unique genes, were phenotypically characterized. These *C. glabrata* mutant strains were grown in YPD medium in 96-well plates for 14–16 h, OD_600_ was normalized to 1.0 and 5 µl of 150-fold diluted culture was spotted onto different plates. Growth profiles, recorded after 48 h of incubation either at 30°C or 42°C, are color coded and indicated at the bottom. Rows correspond to mutants and columns to different phenotypic tests. The conditions used for profiling were rich medium (YPD), thermal stress (42°C), minimal medium (YNB), tissue culture medium (RPMI medium), tissue culture medium (RPMI medium) containing 10% serum, ER stress (10 mM dithiothreitol), oxidative stress (20 mM hydrogen peroxide), replication stress (50 mM hydroxyurea), genotoxic stress (25 µM camptothecin) salt stress (500 mM sodium chloride), iron starvation (200 µM 2,2′-dipyridyl) cell wall stress (10 mM caffeine), membrane stress (0.005% sodium dodecyl sulfate), antifungal stress (16 µg/ml fluconazole), low pH (pH 2.0), neutral pH (pH 7.0). Scaled expression values are colour-coded according to the legend at the bottom. (**B**) Single-strain infections of PMA-activated THP-1 cells to assess the number of intracellular yeasts for mutants defective in chromatin organization. THP-1 macrophages were lysed 2 h and 24 h post infection and cell lysates were plated onto YPD medium to enumerate the viable *C. glabrata* cells. Increase in CFUs for each strain was determined by dividing the CFUs obtained at 24 h with those for 2 h. Survival ratio represent the CFU ratio of mutant/wild type after 24 h of infection. (**C**) Growth curve analysis of *wt* and indicated mutants in YPD medium at 30°C. Absorbance at 600 nm was monitored over a 42 h time course at indicated time intervals. Data are represented as mean values of three independent growth analyses. (**D**) Growth curve analysis of *wt* and indicated mutants in RPMI medium supplemented with 10% serum at 37°C. Absorbance at 600 nm was monitored over a 48 h time course at indicated time intervals. Data are represented as mean values of three independent growth analyses. (**E**) Equal volume of 10-fold serial dilutions of *wt* and mutant cultures was spotted onto YPD plates containing different stress agents at the following concentrations and growth was scored after 48 h: methylmethane sulfonate (MMS; 0.03%), camptothecin (CPT; 25 µM), hydroxyurea (HU; 50 mM) and hydrogen peroxide (H_2_O_2_, 20 mM).
(TIF)

Figure S3 **Chromatin of 24 h macrophage-internalized** ***C. glabrata*** **cells is sensitive to micrococcal nuclease digestion.** Chromatin extracted from cells grown either in RPMI or incubated with activated THP1-cells was treated with MNase at 10 units/ml for 15 min and 100 ng digested samples were resolved by agarose gel electrophoresis.
(TIF)

Figure S4 **Macrophage-internalized** ***C. glabrata*** **cells display altered epigenetic signature.** Densitometry of three to five independent Western blots, performed on *wt* whole-cell extracts with antibodies against indicated proteins and modifications, was used to quantify the ratios of histones (A) and histone post-translational modifications (B) in macrophage-internalized yeasts to those in RPMI-grown yeasts and data are plotted as fold change ± SEM in arbitrary units (AUs). Densitometric quantification of ratios of modified (acetylated/methylated/phosphorylated) histone to total histone levels in macrophage-internalized *C. glabrata* cells is tabulated and presented as % change in PTMs (C). Ratio of dimethylation of histone H3 at lysine 27 in macrophage-ingested cells to that in RPMI-cultured yeasts (D). ImageJ software was used to quantify bands.
(TIF)

Figure S5 ***Cgrsc3-aΔ*** **,** ***Cgrsc3-bΔ*** **,** ***Cgrsc3-aΔbΔ*** **,** ***Cgrtt107Δ*** **,** ***Cgrtt109Δ*** **, and** ***Cgsgs1Δ*** **, mutants displayed growth profiles similar to the wild-type.** (**A**) Growth curve analysis of *wt* and indicated deletion strains in YPD medium at 30°C. Absorbance at 600 nm was monitored over a 48 h time course at indicated time intervals. Data are represented as mean values of three independent growth analyses. (**B**) Growth curve analysis of *wt* and indicated deletion strains in RPMI medium at 37°C. Data are represented as mean values of three independent growth analyses. (**C**) Ectopic expression of *CgRTT107* and *CgRTT109* complement the sensitivity of *Cgrtt107Δ* and *Cgrtt109Δ* mutants to DNA damage-causing agents. Growth profiles of wild-type (*wt*), *Cgrtt107Δ* and *Cgrtt109Δ* strains harboring either empty vector (pRK74) or plasmid expressing *CgRTT107* (pRK700) or *CgRTT109* (pRK941) from *PGK1* promoter in the presence of methylmethane sulfonate (MMS; 0.03%) and camptothecin (CPT; 25 µM) were recorded after 2 days of growth at 30°C. (**D**) Growth curve analysis of *wt*, *Cgrtt107Δ* and *Cgrtt109Δ* strains in YPD and YPD medium containing 20 mM H_2_O_2_ at 30°C. Absorbance at 600 nm was monitored over a 48 h time course at indicated time intervals. Data are represented as mean values of three independent growth analyses. (**E**) Chromatin of *Cgrsc3-aΔ*, *Cgrsc3-aΔ bΔ* and *Cgrtt109Δ* mutants display reduced sensitivity to micrococcal nuclease digestion. Chromatin extracted from cells grown in YPD medium were treated with MNase at 10 units/ml for 15 min and 200 ng digested samples were resolved by agarose gel electrophoresis. (**F**) *Cgrtt109Δ* cells displayed residual acetylation on lysine 56 of histone H3. Immunoblot analysis on whole-cell extracts of wild-type, *Cgrtt109Δ* and *Cgrtt109Δ/CgRTT109* (reconstituted mutant) strains with antibodies against indicated proteins/modifications.
(TIF)

Figure S6 **Differential chromatin architecture of macrophage-ingested** ***Cgrsc3-aΔ*** **,** ***Cgrtt107Δ*** **, and** ***Cgrtt109Δ*** **mutant cells.** Densitometry of three to five independent Western blots, performed on whole-cell extracts of *Cgrsc3-aΔ* (A), *Cgrtt109Δ* (B) and *Cgrtt107Δ* (C) mutants with antibodies against indicated proteins and modifications, was used to quantify the ratios of histones and histone post-translational modifications in macrophage-internalized yeasts to those in RPMI-grown yeasts and data are plotted as fold change ± SEM in arbitrary units (AUs). Densitometric quantification of ratios of modified (acetylated/methylated/phosphorylated) histone to total histone levels in macrophage-internalized mutant cells is tabulated and presented as % change in PTMs. ImageJ software was used to quantify bands.
(TIF)

Figure S7 **Differential patterns of gene expression in** ***C. glabrata*** **cells upon macrophage ingestion.** Heat maps of expression levels of clustered and differentially expressed genes, belonging to the GO category of protein modification (A), carbohydrate metabolic process (B), cellular amino acid metabolism (C) and vesicle-mediated transport (D) in macrophage-internalized *wt* (1), *Cgrsc3-aΔ* (2) and *Cgrtt109Δ* (3) cells compared to the corresponding RPMI-grown cells. Lanes 4–7 represent the expression of differentially expressed genes in macrophage-internalized *Cgrsc3-aΔ* (4) and *Cgrtt109Δ* (5) and RPMI-grown *Cgrsc3-aΔ* (6) and *Cgrtt109Δ* (7) compared to the macrophage-internalized and RPMI-cultured *wt* cells, respectively.
(TIF)

Figure S8 **GO Slim Mapper analysis of differentially expressed genes (p-value≤0.05) in** ***wt*** **,** ***Cgrsc3-aΔ*** **and** ***Cgrtt109Δ*** **cells upon macrophage internalization.** A set of 100, 219, and 346 up-regulated (A) and 114, 213, and 337 down-regulated (B) genes in *wt*, *Cgrsc3-aΔ* and *Cgrtt109Δ* cells, respectively, were functionally annotated *via* gene ontology analysis performed with Slim Mapper at CGD (http://www.candidagenome.org/cgi-bin/GO/goTermMapper). Genes constituting ≥3% of total differentially-regulated gene sets in either of the three strains, *wt*, *Cgrsc3-aΔ* and *Cgrtt109Δ*, are presented with associated-GO process.
(TIF)

Figure S9 **Transcriptional profiling analysis of chromatin organization defective mutants.** **A–B:** Venn diagram illustrating the overlap between up-regulated (A) and down-regulated (B) genes in the macrophage-internalized *Cgrsc3-aΔ* and *Cgrtt109Δ* cells compared to the macrophage-internalized *wt* cells. **C–E:** Quantitative RT-PCR confirmation of transcript-fold changes from microarray expression profiling. PMA-activated THP-1 cells were infected with *C. glabrata* cells at 1∶10 MOI, washed thrice with PBS after 2 h and lysed in water to recover internalized yeasts 10 h post-infection. qRT-PCR analyses of indicated genes were performed in duplicate with SYBR Green dye using ABI PRISM 7500 Sequence Detection System. mRNA levels in 10 h RPMI-grown *Cgrsc3-aΔ* and *Cgrtt109Δ* mutants were compared to the 10 h RPMI-cultured wild-type cells to validate the changes in the transcript levels owing to the disruption of *CgRSC3-A* and *CgRTT109* genes. Data were normalized to an internal *CgGAPDH* mRNA control, and the relative changes in transcriptional level in response to macrophage internalization (C), *CgRSC3-A* (D), *CgRTT109* (E) disruption, were calculated as a ratio of transcript levels of experimental samples versus control samples using the 2^−ΔΔCT^ method. Data represent the means of 3 independent experiments ± SEM (p-value≤0.05). **F:** Logo representation of the DNA-binding sequence of CgRsc3 identified by MEME among the differentially expressed genes in response to macrophage internalization.
(TIF)

Figure S10 **Chromatin remodeling defective mutants are impaired in the utilization of alternative carbon sources.** (**A**) Mutants defective in chromatin organization are impaired to varied extents in the utilization of alternative carbon sources. Equal volume of 10-fold serial dilutions of wild-type and mutant cultures was spotted onto YNB medium containing oleic acid, sodium acetate, lactic acid and citric acid as sole carbon sources and growth was scored after 5–8 days of growth at 30°C. (**B**) Growth curve analysis for *wt* and indicated mutants in YNB medium supplemented with dextrose at 30°C. Absorbance at 600 nm was monitored over a period of 48 h at indicated time intervals. Data are represented as mean values (± SEM) of three independent growth analyses. (**C**) Growth curve analysis for *wt* and indicated mutants in YNB medium supplemented with sodium acetate at 30°C. Absorbance at 600 nm was monitored over a period of 192 h at indicated time intervals. Data are represented as mean values (± SEM) of three independent growth analyses. (**D**) Growth curve analysis for *wt* and indicated mutants in YNB medium supplemented with lactic acid at 30°C. Absorbance at 600 nm was monitored over a period of 120 h at indicated time intervals. Data are represented as mean values (± SEM) of three independent growth analyses. (**E**) Immunoblot analysis on whole-cell extracts of wild-type cells grown for 6 h in YNB containing either 2% dextrose (YNB-D) or 2% sodium acetate (YNB-S) as carbon source with antibodies against indicated proteins/modifications. (**F**) *C. glabrata* wild-type cells exhibit increased lysine deacetylase activity upon growth in medium containing sodium acetate as sole carbon source. Cellular lysine deacetylase activity was measured using trifluoroacetyl-lysine as a substrate. Treatment with 10 nM trichostatin A (TSA) brought the KDAC activity to basal levels, thereby, validating the specificity of the assay.
(TIF)

Table S1 **Summary of the STM screen.**
(DOCX)

Table S2 **Mutants identified in the STM screen for reduced survival in human THP-1 macrophages.**
(DOCX)

Table S3 **List of strains and plasmids used in the study.**
(DOCX)

Table S4 **List of antibodies used in the study.**
(DOCX)

Table S5 **Primers used in the study.**
(DOCX)

Text S1 **Materials and Methods and References for Supplementary Information.**
(DOC)

Text S2 **Quantile normalized microarray data.**
(XLSX)
