# Supplementary figures and images for: Functional Genomic Analysis of Candida glabrata-Macrophage Interaction: Role of Chromatin Remodeling in Virulence
Source: PLoS Pathog. 2012 Aug 16;8(8):e1002863. doi: 10.1371/journal.ppat.1002863 (PMC3420920; doi:10.1371/journal.ppat.1002863)

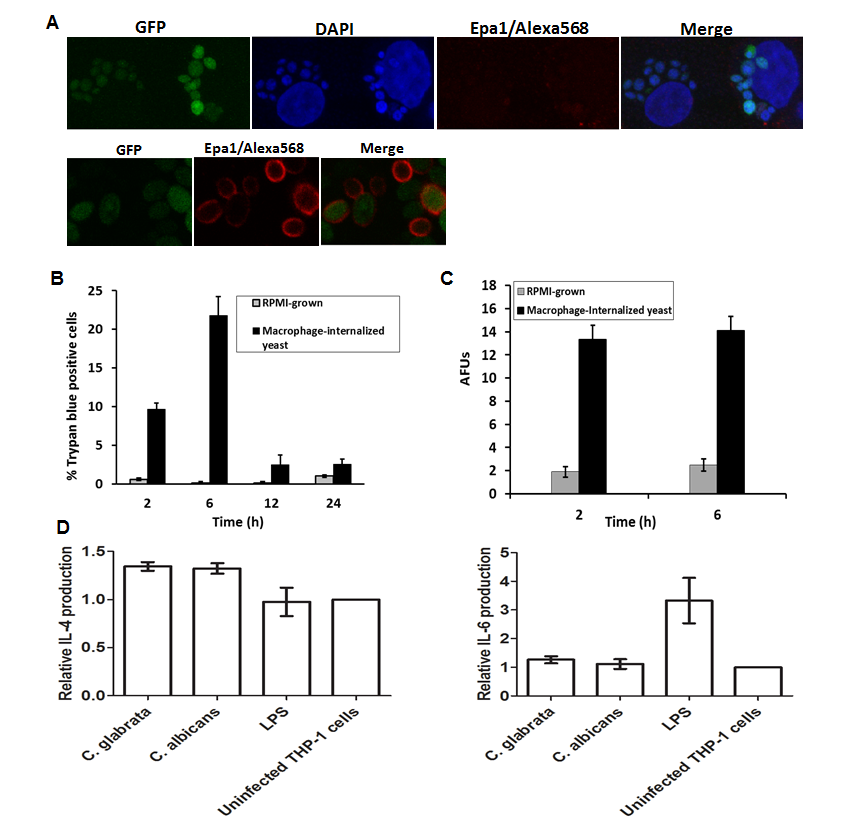

Supplement: Figure S1 — C. glabrata cells are killed by activated THP1-cells. (A) Inside/Outside staining to validate intracellular replication of C. glabrata wild-type cells. After 24 h of infection, GFP-expressing C. glabrata cells were labeled with anti-Epa1 (Epithelial adhesin 1) antibody and visualized using Alexa Fluor 568-conjugated secondary antibody. Owing to inaccessibility to primary and secondary antibody, intracellular yeast fluoresced green. No doubly-labelled (red and green) extracellular yeasts were observed. The bottom panel serves as a control and confirms the reactivity of anti-Epa1 antibody in RPMI-grown C. glabrata wild-type cells. Epa1 being a cell surface protein, a clear distinct ring of surface-localized Epa1 was observed. (B) Trypan blue exclusion assay to assess the viability of RPMI-grown and macrophage-internalized C. glabrata cells in PMA-activated THP-1 cells. Cells were collected at indicated time points and stained with 0.4% trypan blue for 10 min. A minimum of total 300 cells (stained (dead) and unstained (viable)) were counted microscopically for data point. Cell viability data were plotted as the percentage of trypan blue positive cells and represent the mean of three independent analyses (± SEM). (C) Measurement of ROS levels in macrophage-internalized yeast at indicated time points post infection using the DCF (2′,7′-dichlorofluorescein) fluorescence assay. Data are from three independent analyses ± SEM. AFUs = Arbitrary fluorescence units. (D) C. glabrata elicits interleukin-4 (IL-4) production in PMA-activated THP-1 cells. PMA-activated THP1 cells infected with yeast cells at a MOI of 1∶10. Two hours post infection, cells were washed with PBS thrice and incubated in fresh RPMI medium at 37°C. As a control, THP-1 cells were treated with 1 µg/ml LPS for 24 h. After 24 h, supernatants were collected, centrifuged at 3000 rpm for 5 min to remove any particulate matter if any. Expression of indicated cytokines was enumerated using BD OptEA ELISA kit as per [file ppat.1002863.s001.tif]

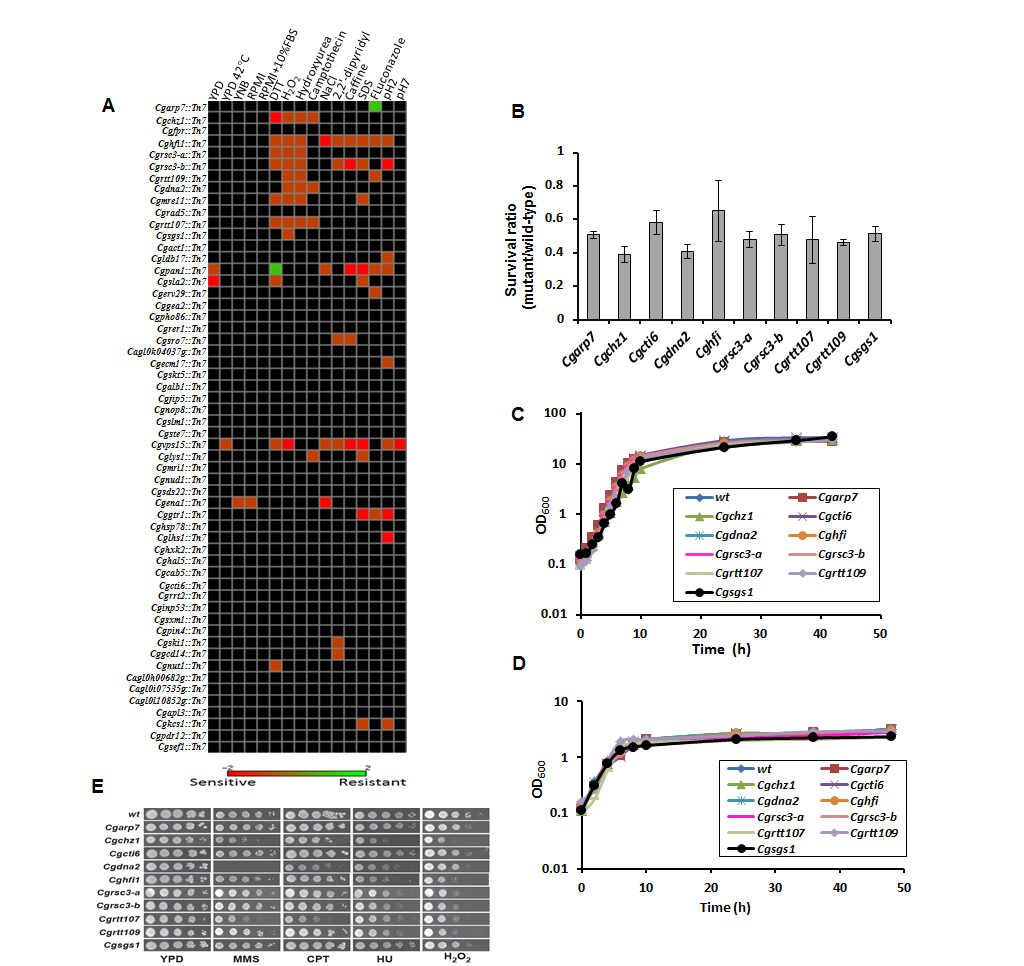

Supplement: Figure S2 — Mutants disrupted for chromatin organization display reduced survival in macrophages and varied sensitivity to genotoxic stress. (A) Heat map depicting the growth of mutants, identified through the STM screen, in the presence of diverse stresses. Since several mutants with multiple Tn7 insertions in the same gene and twenty mutants harboring Tn7 insertions in intergenic regions were identified in the STM screen, all these mutants were not subjected to further analysis. Instead, a total of 56 mutants carrying Tn7 insertions in unique genes, were phenotypically characterized. These C. glabrata mutant strains were grown in YPD medium in 96-well plates for 14–16 h, OD600 was normalized to 1.0 and 5 µl of 150-fold diluted culture was spotted onto different plates. Growth profiles, recorded after 48 h of incubation either at 30°C or 42°C, are color coded and indicated at the bottom. Rows correspond to mutants and columns to different phenotypic tests. The conditions used for profiling were rich medium (YPD), thermal stress (42°C), minimal medium (YNB), tissue culture medium (RPMI medium), tissue culture medium (RPMI medium) containing 10% serum, ER stress (10 mM dithiothreitol), oxidative stress (20 mM hydrogen peroxide), replication stress (50 mM hydroxyurea), genotoxic stress (25 µM camptothecin) salt stress (500 mM sodium chloride), iron starvation (200 µM 2,2′-dipyridyl) cell wall stress (10 mM caffeine), membrane stress (0.005% sodium dodecyl sulfate), antifungal stress (16 µg/ml fluconazole), low pH (pH 2.0), neutral pH (pH 7.0). Scaled expression values are colour-coded according to the legend at the bottom. (B) Single-strain infections of PMA-activated THP-1 cells to assess the number of intracellular yeasts for mutants defective in chromatin organization. THP-1 macrophages were lysed 2 h and 24 h post infection and cell lysates were plated onto YPD medium to enumerate the viable C. glabrata cells. Increase in CFUs for each strain was determined by dividing the CF [file ppat.1002863.s002.tif]

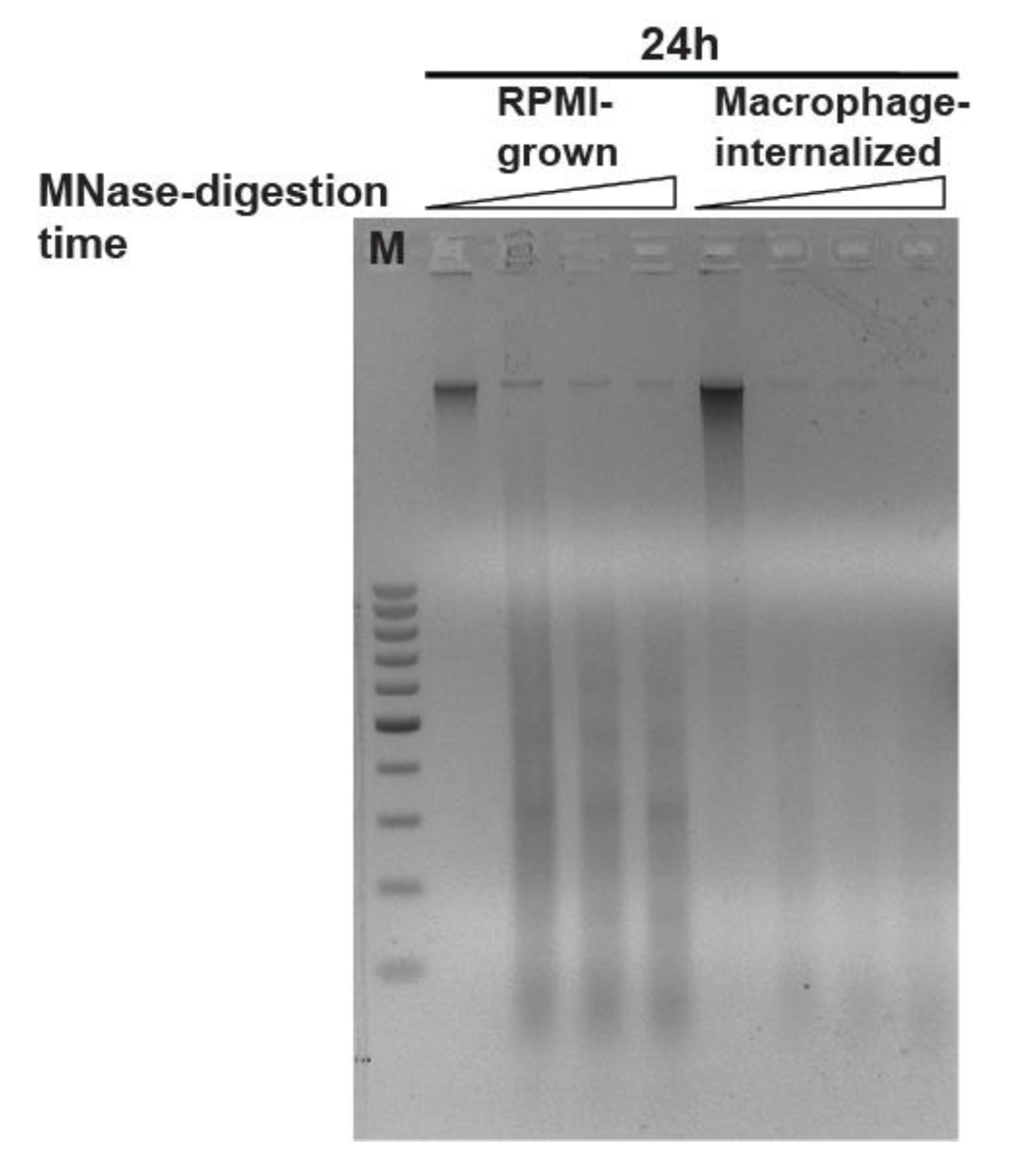

Supplement: Figure S3 — Chromatin of 24 h macrophage-internalized C. glabrata cells is sensitive to micrococcal nuclease digestion. Chromatin extracted from cells grown either in RPMI or incubated with activated THP1-cells was treated with MNase at 10 units/ml for 15 min and 100 ng digested samples were resolved by agarose gel electrophoresis. (TIF) [file ppat.1002863.s003.tif]

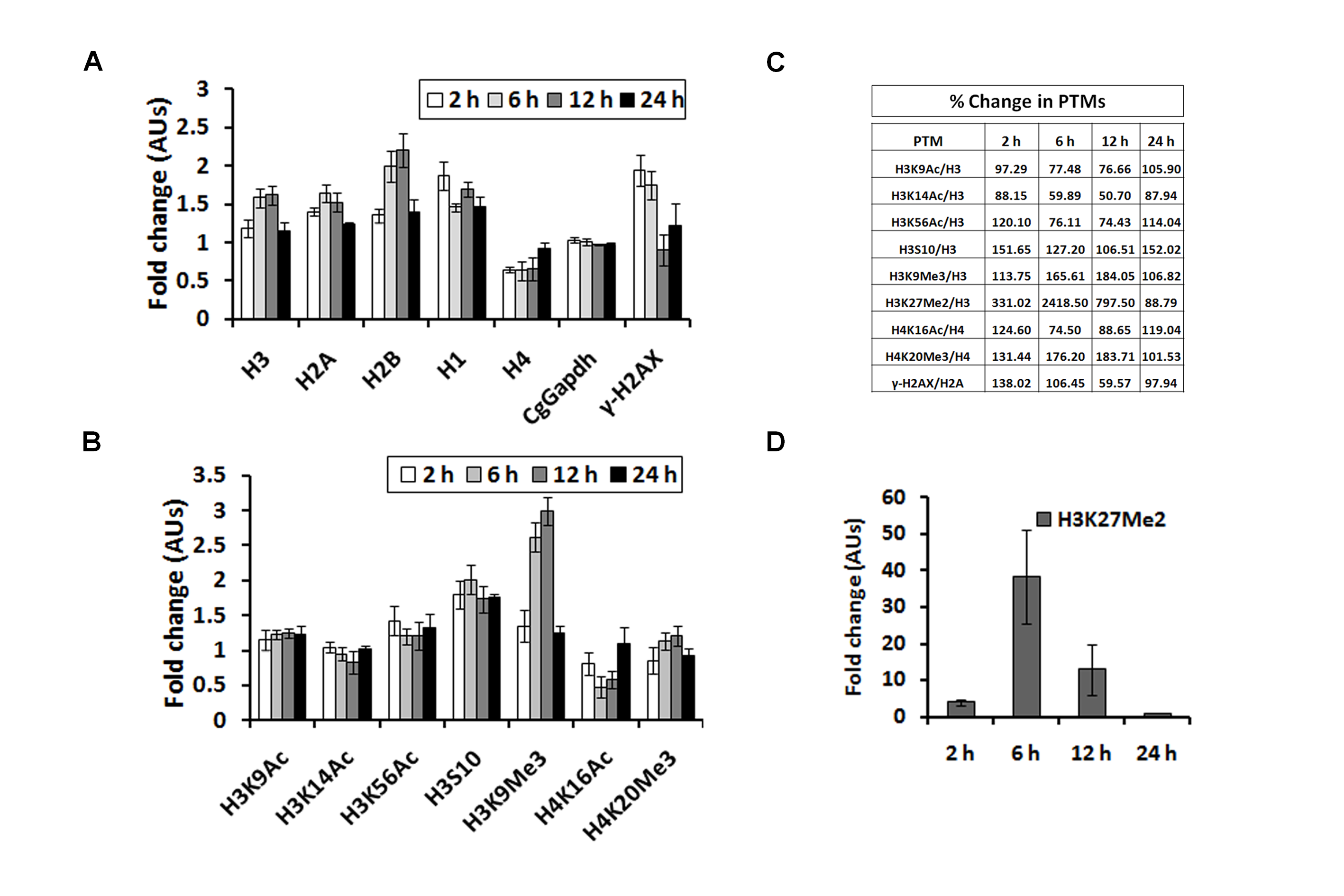

Supplement: Figure S4 — Macrophage-internalized C. glabrata cells display altered epigenetic signature. Densitometry of three to five independent Western blots, performed on wt whole-cell extracts with antibodies against indicated proteins and modifications, was used to quantify the ratios of histones (A) and histone post-translational modifications (B) in macrophage-internalized yeasts to those in RPMI-grown yeasts and data are plotted as fold change ± SEM in arbitrary units (AUs). Densitometric quantification of ratios of modified (acetylated/methylated/phosphorylated) histone to total histone levels in macrophage-internalized C. glabrata cells is tabulated and presented as % change in PTMs (C). Ratio of dimethylation of histone H3 at lysine 27 in macrophage-ingested cells to that in RPMI-cultured yeasts (D). ImageJ software was used to quantify bands. (TIF) [file ppat.1002863.s004.tif]

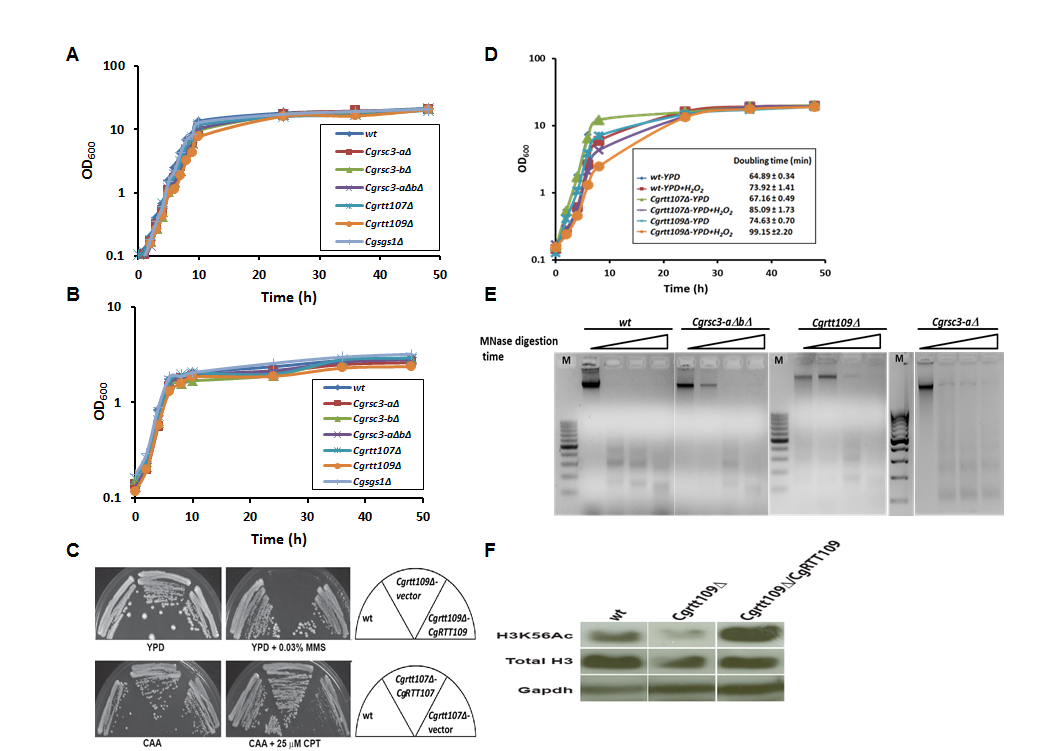

Supplement: Figure S5 — Cgrsc3-aΔ , Cgrsc3-bΔ , Cgrsc3-aΔbΔ , Cgrtt107Δ , Cgrtt109Δ , and Cgsgs1Δ , mutants displayed growth profiles similar to the wild-type. (A) Growth curve analysis of wt and indicated deletion strains in YPD medium at 30°C. Absorbance at 600 nm was monitored over a 48 h time course at indicated time intervals. Data are represented as mean values of three independent growth analyses. (B) Growth curve analysis of wt and indicated deletion strains in RPMI medium at 37°C. Data are represented as mean values of three independent growth analyses. (C) Ectopic expression of CgRTT107 and CgRTT109 complement the sensitivity of Cgrtt107Δ and Cgrtt109Δ mutants to DNA damage-causing agents. Growth profiles of wild-type (wt), Cgrtt107Δ and Cgrtt109Δ strains harboring either empty vector (pRK74) or plasmid expressing CgRTT107 (pRK700) or CgRTT109 (pRK941) from PGK1 promoter in the presence of methylmethane sulfonate (MMS; 0.03%) and camptothecin (CPT; 25 µM) were recorded after 2 days of growth at 30°C. (D) Growth curve analysis of wt, Cgrtt107Δ and Cgrtt109Δ strains in YPD and YPD medium containing 20 mM H2O2 at 30°C. Absorbance at 600 nm was monitored over a 48 h time course at indicated time intervals. Data are represented as mean values of three independent growth analyses. (E) Chromatin of Cgrsc3-aΔ, Cgrsc3-aΔ bΔ and Cgrtt109Δ mutants display reduced sensitivity to micrococcal nuclease digestion. Chromatin extracted from cells grown in YPD medium were treated with MNase at 10 units/ml for 15 min and 200 ng digested samples were resolved by agarose gel electrophoresis. (F) Cgrtt109Δ cells displayed residual acetylation on lysine 56 of histone H3. Immunoblot analysis on whole-cell extracts of wild-type, Cgrtt109Δ and Cgrtt109Δ/CgRTT109 (reconstituted mutant) strains with antibodies against indicated proteins/modifications. (TIF) [file ppat.1002863.s005.tif]

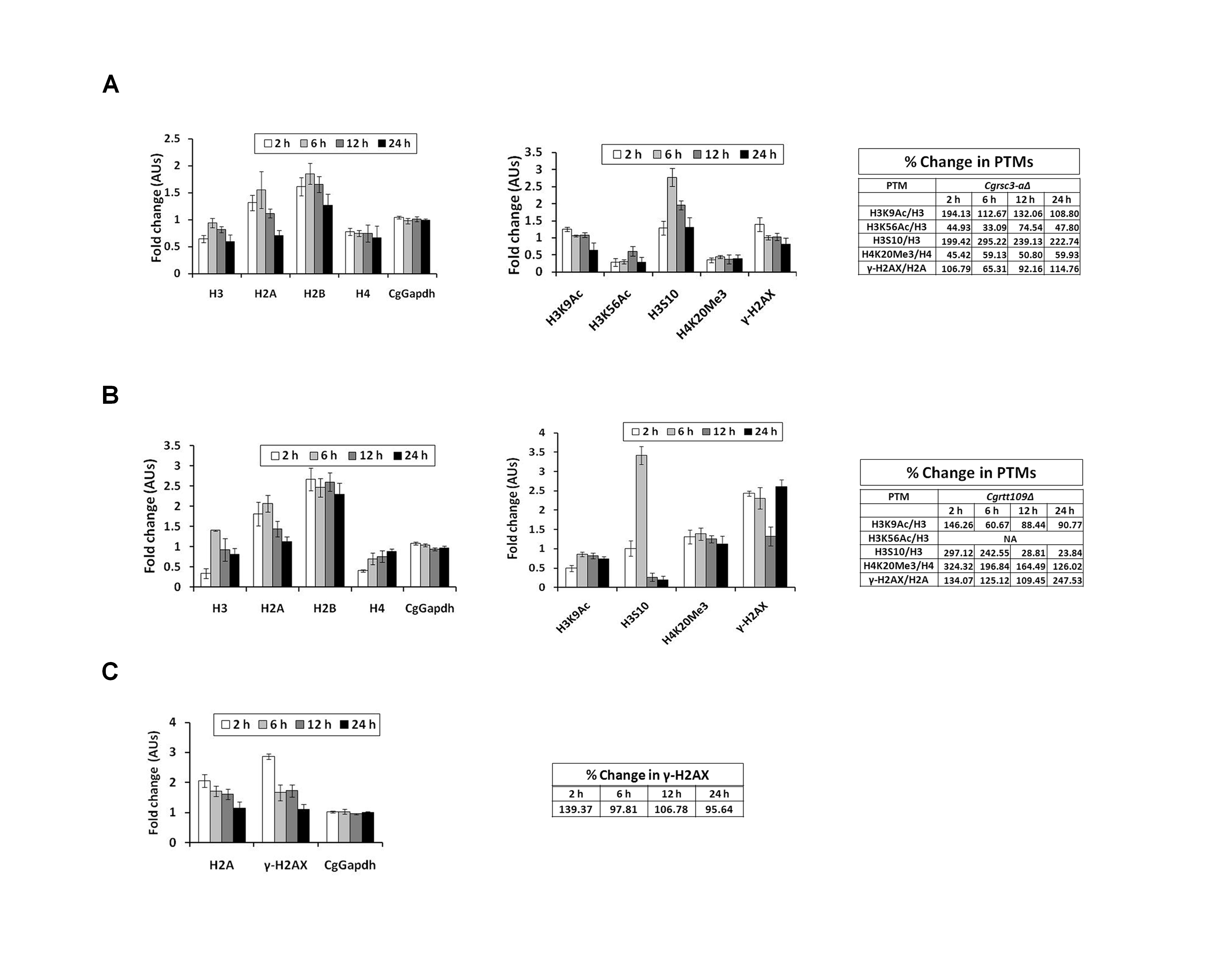

Supplement: Figure S6 — Differential chromatin architecture of macrophage-ingested Cgrsc3-aΔ , Cgrtt107Δ , and Cgrtt109Δ mutant cells. Densitometry of three to five independent Western blots, performed on whole-cell extracts of Cgrsc3-aΔ (A), Cgrtt109Δ (B) and Cgrtt107Δ (C) mutants with antibodies against indicated proteins and modifications, was used to quantify the ratios of histones and histone post-translational modifications in macrophage-internalized yeasts to those in RPMI-grown yeasts and data are plotted as fold change ± SEM in arbitrary units (AUs). Densitometric quantification of ratios of modified (acetylated/methylated/phosphorylated) histone to total histone levels in macrophage-internalized mutant cells is tabulated and presented as % change in PTMs. ImageJ software was used to quantify bands. (TIF) [file ppat.1002863.s006.tif]

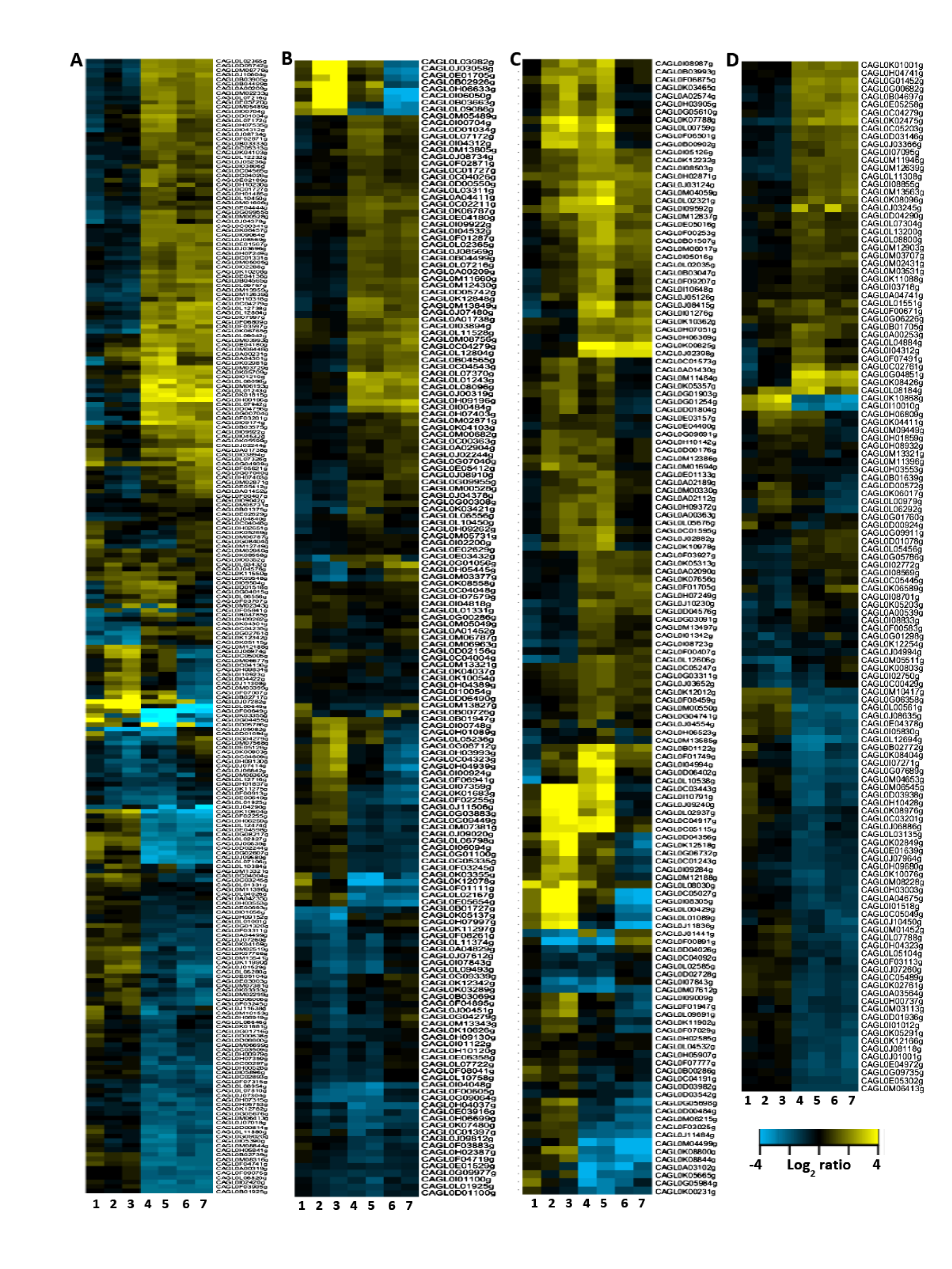

Supplement: Figure S7 — Differential patterns of gene expression in C. glabrata cells upon macrophage ingestion. Heat maps of expression levels of clustered and differentially expressed genes, belonging to the GO category of protein modification (A), carbohydrate metabolic process (B), cellular amino acid metabolism (C) and vesicle-mediated transport (D) in macrophage-internalized wt (1), Cgrsc3-aΔ (2) and Cgrtt109Δ (3) cells compared to the corresponding RPMI-grown cells. Lanes 4–7 represent the expression of differentially expressed genes in macrophage-internalized Cgrsc3-aΔ (4) and Cgrtt109Δ (5) and RPMI-grown Cgrsc3-aΔ (6) and Cgrtt109Δ (7) compared to the macrophage-internalized and RPMI-cultured wt cells, respectively. (TIF) [file ppat.1002863.s007.tif]

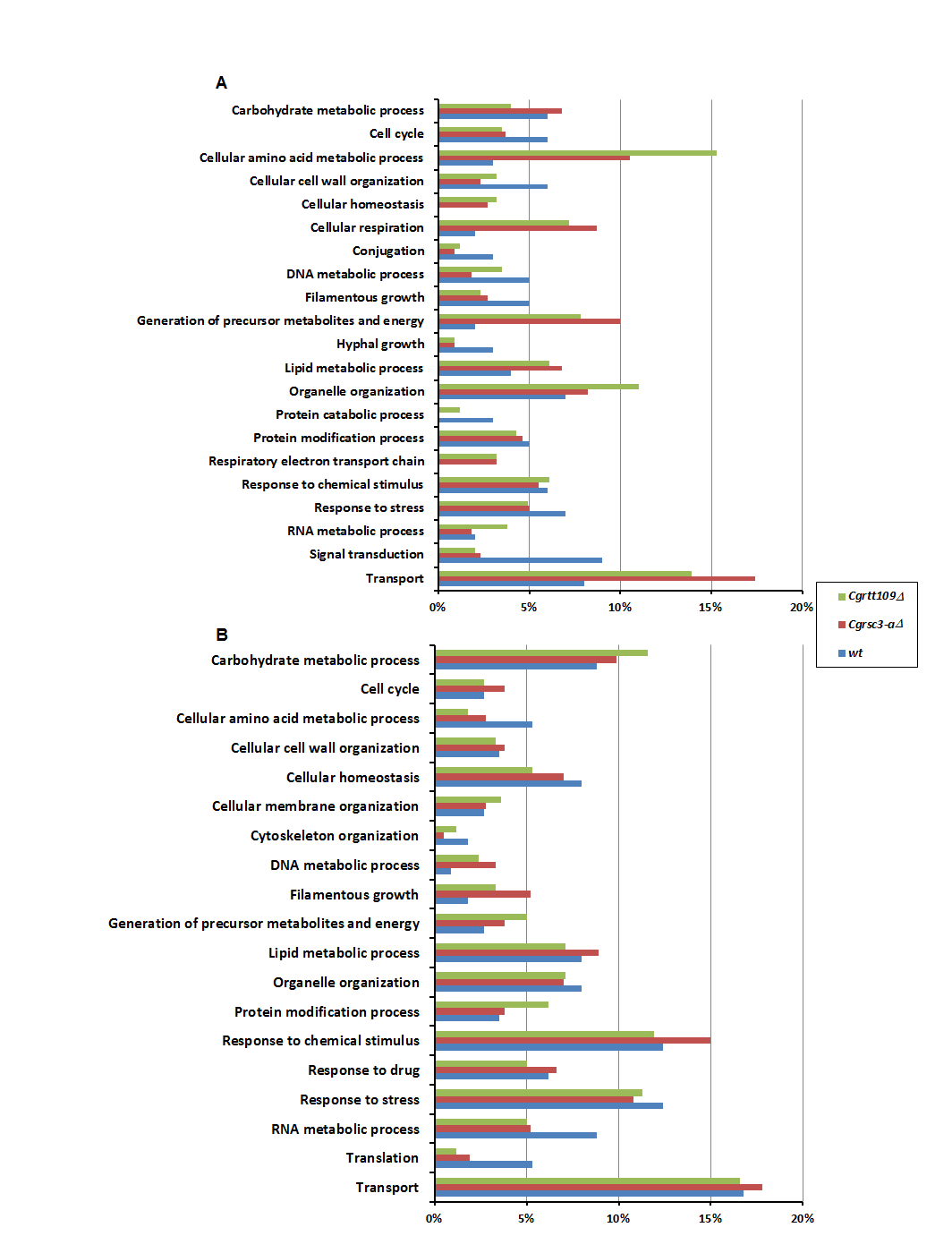

Supplement: Figure S8 — GO Slim Mapper analysis of differentially expressed genes (p-value≤0.05) in wt , Cgrsc3-aΔ and Cgrtt109Δ cells upon macrophage internalization. A set of 100, 219, and 346 up-regulated (A) and 114, 213, and 337 down-regulated (B) genes in wt, Cgrsc3-aΔ and Cgrtt109Δ cells, respectively, were functionally annotated via gene ontology analysis performed with Slim Mapper at CGD (http://www.candidagenome.org/cgi-bin/GO/goTermMapper). Genes constituting ≥3% of total differentially-regulated gene sets in either of the three strains, wt, Cgrsc3-aΔ and Cgrtt109Δ, are presented with associated-GO process. (TIF) [file ppat.1002863.s008.tif]

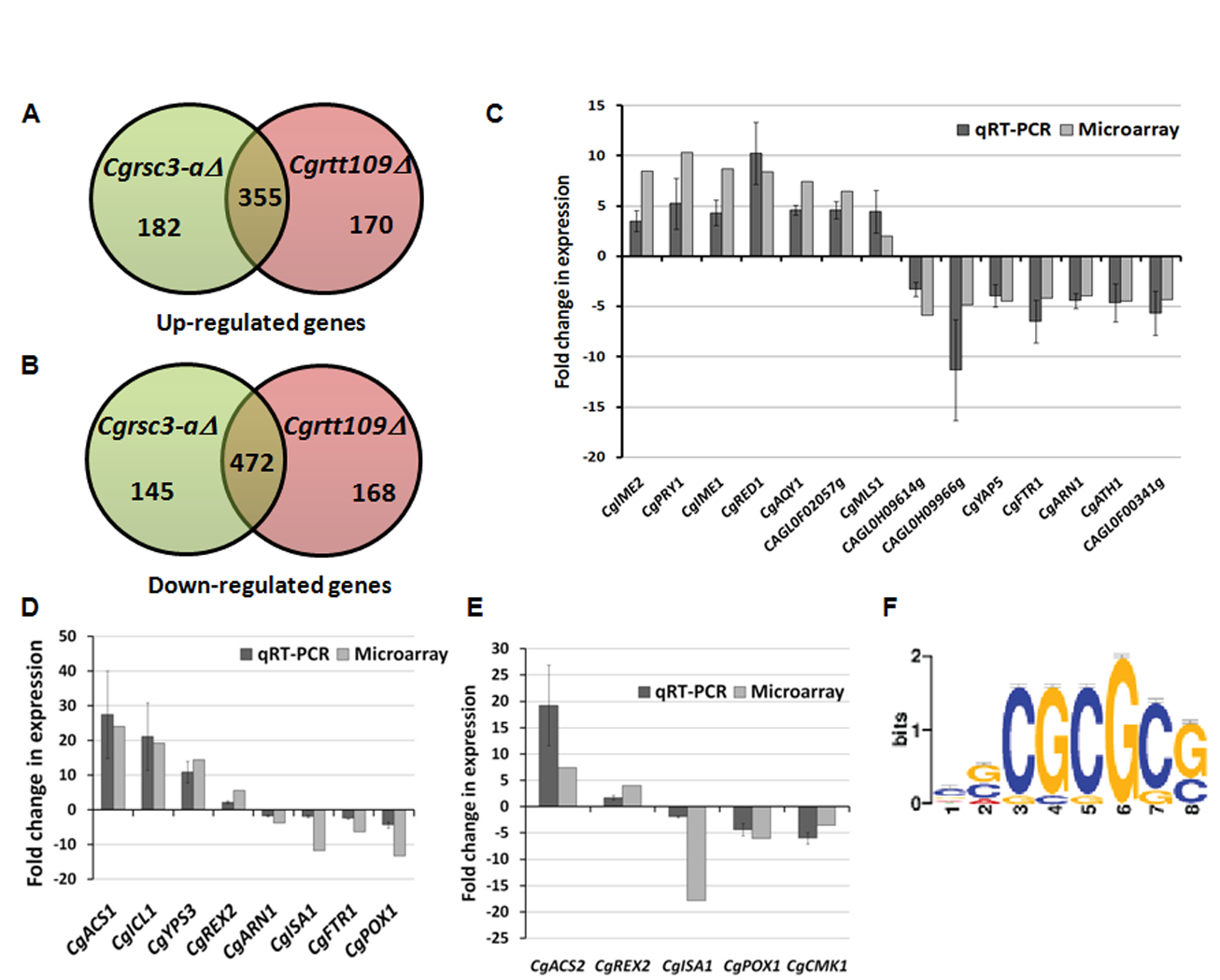

Supplement: Figure S9 — Transcriptional profiling analysis of chromatin organization defective mutants. A–B: Venn diagram illustrating the overlap between up-regulated (A) and down-regulated (B) genes in the macrophage-internalized Cgrsc3-aΔ and Cgrtt109Δ cells compared to the macrophage-internalized wt cells. C–E: Quantitative RT-PCR confirmation of transcript-fold changes from microarray expression profiling. PMA-activated THP-1 cells were infected with C. glabrata cells at 1∶10 MOI, washed thrice with PBS after 2 h and lysed in water to recover internalized yeasts 10 h post-infection. qRT-PCR analyses of indicated genes were performed in duplicate with SYBR Green dye using ABI PRISM 7500 Sequence Detection System. mRNA levels in 10 h RPMI-grown Cgrsc3-aΔ and Cgrtt109Δ mutants were compared to the 10 h RPMI-cultured wild-type cells to validate the changes in the transcript levels owing to the disruption of CgRSC3-A and CgRTT109 genes. Data were normalized to an internal CgGAPDH mRNA control, and the relative changes in transcriptional level in response to macrophage internalization (C), CgRSC3-A (D), CgRTT109 (E) disruption, were calculated as a ratio of transcript levels of experimental samples versus control samples using the 2−ΔΔCT method. Data represent the means of 3 independent experiments ± SEM (p-value≤0.05). F: Logo representation of the DNA-binding sequence of CgRsc3 identified by MEME among the differentially expressed genes in response to macrophage internalization. (TIF) [file ppat.1002863.s009.tif]

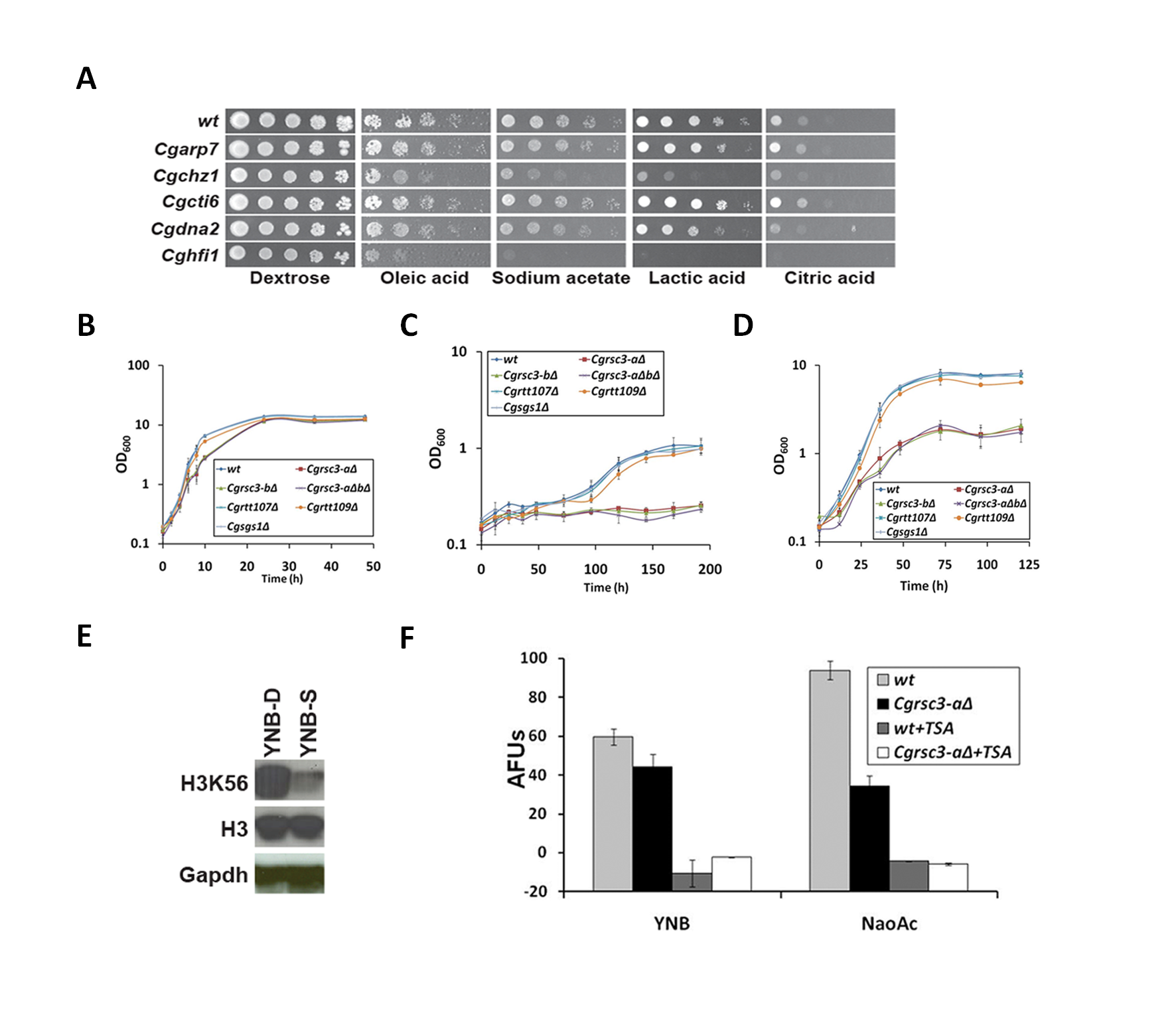

Supplement: Figure S10 — Chromatin remodeling defective mutants are impaired in the utilization of alternative carbon sources. (A) Mutants defective in chromatin organization are impaired to varied extents in the utilization of alternative carbon sources. Equal volume of 10-fold serial dilutions of wild-type and mutant cultures was spotted onto YNB medium containing oleic acid, sodium acetate, lactic acid and citric acid as sole carbon sources and growth was scored after 5–8 days of growth at 30°C. (B) Growth curve analysis for wt and indicated mutants in YNB medium supplemented with dextrose at 30°C. Absorbance at 600 nm was monitored over a period of 48 h at indicated time intervals. Data are represented as mean values (± SEM) of three independent growth analyses. (C) Growth curve analysis for wt and indicated mutants in YNB medium supplemented with sodium acetate at 30°C. Absorbance at 600 nm was monitored over a period of 192 h at indicated time intervals. Data are represented as mean values (± SEM) of three independent growth analyses. (D) Growth curve analysis for wt and indicated mutants in YNB medium supplemented with lactic acid at 30°C. Absorbance at 600 nm was monitored over a period of 120 h at indicated time intervals. Data are represented as mean values (± SEM) of three independent growth analyses. (E) Immunoblot analysis on whole-cell extracts of wild-type cells grown for 6 h in YNB containing either 2% dextrose (YNB-D) or 2% sodium acetate (YNB-S) as carbon source with antibodies against indicated proteins/modifications. (F) C. glabrata wild-type cells exhibit increased lysine deacetylase activity upon growth in medium containing sodium acetate as sole carbon source. Cellular lysine deacetylase activity was measured using trifluoroacetyl-lysine as a substrate. Treatment with 10 nM trichostatin A (TSA) brought the KDAC activity to basal levels, thereby, validating the specificity of the assay. (TIF) [file ppat.1002863.s010.tif]
