# Supplementary material for: Functional Genomic Analysis of Candida glabrata-Macrophage Interaction: Role of Chromatin Remodeling in Virulence
Source: PLoS Pathog. 2012 Aug 16;8(8):e1002863. doi: 10.1371/journal.ppat.1002863 (PMC3420920; doi:10.1371/journal.ppat.1002863)
Supplement: Table S1 — Summary of the STM screen. (DOCX) [file ppat.1002863.s011.docx]

**Table S1: Summary of the STM screen**

| Total number of mutants screened | 18,350 |
| --- | --- |
| Mutants selected with a cut-off value of ≤0.1 and ≥6 | 168 |
| Number of Up mutants identified | 35 |
| Number of Down mutants identified | 133 |
| Number of genes identified | 56 |
| Number of mutants with Tn*7* insertion in the intergenic regions | 20 |
