# Supplementary material for: Functional Genomic Analysis of Candida glabrata-Macrophage Interaction: Role of Chromatin Remodeling in Virulence
Source: PLoS Pathog. 2012 Aug 16;8(8):e1002863. doi: 10.1371/journal.ppat.1002863 (PMC3420920; doi:10.1371/journal.ppat.1002863)
Supplement: Table S2 — Mutants identified in the STM screen for reduced survival in human THP-1 macrophages. (DOCX) [file ppat.1002863.s012.docx]

**Table S2: Mutants identified in the STM screen for reduced survival in human THP-1 macrophages**

| **Mutant** | **Op/Ip ratio** | ***CAGL-ORF*** | ***S. cerevisiae* ortholog** | **Description** | **Position of Tn*7* insertion** | **ORF length (nt)** |
| --- | --- | --- | --- | --- | --- | --- |
| **Chromatin organization** | | | | | | |
| *Cgarp7::Tn7* | 0.08 | *CAGL0K05335g* | *ARP7* | Component of both the SWI/SNF and RSC chromatin remodeling complexes | 980 | 1590 |
| *Cgchz1::Tn7* | 0.10 | *CAGL0C02475g* | *CHZ1* | Histone chaperone for Htz1/H2A-H2B dimer | 24 bps from stop codon | 447 |
| *Cgfpr4::Tn7* | 0.03 | *CAGL0M00638g* | *FPR4* | Peptidyl-prolyl cis-trans isomerase | 201 | 1197 |
| *Cghfi1::Tn7* | 0.08 | *CAGL0H00616g* | *HFI1* | Adaptor protein required for structural integrity of the SAGA complex | 1111 | 1356 |
| *Cgrsc3-a::Tn7* | 0.01 | *CAGL0D03850g* | *RSC3* | Component of the RSC chromatin remodeling complex | 76 | 2505 |
| *Cgrsc3-b::Tn7* | 0.10 | *CAGL0H01507g* | *RSC3* | Component of the RSC chromatin remodeling complex | 1024 | 2385 |
| *Cgrtt109::Tn7* | 0.09 | *CAGL0D05786g* | *RTT109* | Histone acetyltransferase | 1268 | 1272 |
| **DNA repair** | | | | | | |
| *Cgdna2::Tn7* | 0.02 | *CAGL0I02222g* | *DNA2* | Essential tripartite DNA replication factor with single-stranded DNA-dependent ATPase, ATP-dependent nuclease, and helicase activities | 3354 | 4539 |
| *Cgmre11::Tn7* | 0.05 | *CAGL0M11220g* | *MRE11* | Subunit of a complex with Rad50 and Xrs2 (MRX complex) | 1447 | 1971 |
| *Cgrad5::Tn7* | 0.11 | *CAGL0A03432g* | *RAD5* | DNA helicase | 2525 | 3456 |
| *Cgrtt107::Tn7* | 0.07 | *CAGL0I01958g* | *RTT107* | Protein implicated in Mms22- dependent DNA repair during S phase | 1429 | 3300 |
| *Cgsgs1::Tn7* | 0.03 | *CAGL0H00759g* | *SGS1* | Nucleolar DNA helicase of the RecQ family involved in maintenance of genome integrity | 1485 | 2157 |
| **Golgi vesicle transport** | | | | | | |
| *Cgerv29::Tn7* | 0.04 | *CAGL0G00176g* | *ERV29* | Protein localized to COPII-coated vesicles | 393 | 960 |
| *Cggea2::Tn7* | 0.06 | *CAGL0M08052g* | *GEA2* | Guanine nucleotide exchange factor for ADP ribosylation factors | 1260 | 3933 |
| *Cgpho86::Tn7* | 0.03 | *CAGL0L05456g* | *PHO86* | Endoplasmic reticulum resident protein | 245 | 921 |
| *Cgrer1::Tn7* | 0.05 | *CAGL0C01837g* | *RER1* | Protein involved in retention of membrane proteins, including Sec12, in the ER | 1640 | 1925 |
| *Cgsro7::Tn7* | 0.10 | *CAGL0K05291g* | *SRO7* | Effector of RabGTPase Sec4 | 1385 | 3147 |
| **Endocytosis** | | | | | | |
| *Cgact1::Tn7* | 0.03 | *CAGL0K12694g* | *ACT1* | Actin, structural protein | 328 | 2007 |
| *Cgldb17::Tn7* | 0.03 | *CAGL0I03696g* | *LDB17* | Protein of unknown function | 702 | 1434 |
| *Cgpan1::Tn7* | 0.02 | *CAGL0J01892g* | *PAN1* | Part of actin cytoskeleton-regulatory complex Pan1-Sla1-End3 | 698 | 4125 |
| *Cgsla2::Tn7* | 0.03 | *CAGL0J07656g* | *SLA2* | Transmembrane actin-binding protein | 1848 | 2859 |
| **Cell wall organization** | | | | | | |
| *cagl0k04037g::Tn7* | 0.08 | *CAGL0K04037g* |  | 1, 3 β-glucan synthase | 4645 | 5694 |
| *Cgecm17::Tn7* | 0.07 | *CAGL0M00374g* | *ECM17* | ECM17 Sulfite reductase β-subunit | 3346 | 4209 |
| *Cgskt5::Tn7* | 0.10 | *CAGL0A04411g* | *SKT5* | Activator of Chs3 (chitin synthase-III) | 687 | 1968 |
| **Ribosomal large subunit biogenesis** | | | | | | |
| *Cgalb1::Tn7* | 0.10 | *CAGL0L05500g* | *ALB1* | Shuttling pre-60S factor | 419 | 489 |
| *Cgjip5::Tn7* | 0.01 | *CAGL0I06490g* | *JIP5* | Essential protein of unknown function | 790 | 1527 |
| *Cgnop8::Tn7* | 0.02 | *CAGL0B01397g* | *NOP8* | Nucleolar protein required for 60S ribosomal subunit biogenesis | 507 | 1545 |
| **Signaling** | | | | | | |
| *Cgslm1::Tn7* | 0.03 | *CAGL0G02827g* | *SLM1* | Phosphoinositide PI4,5P(2) binding protein | 1327 | 2250 |
| *Cgste7::Tn7* | 0.07 | *CAGL0I03498g* | *STE7* | Signal transducing MAP kinase kinase | 424 | 1356 |
| *Cgvps15::Tn7* | 0.03 | *CAGL0H08437g* | *VPS15* | Myristoylated serine/threonine protein kinase involved in vacuolar protein sorting | 812 | 4233 |
| **Cellular amino acid metabolic process** | | | | | | |
| *Cglys1::Tn7* | 0.01 | *CAGL0F06875g* | *LYS1* | Saccharopine dehydrogenase (NAD+, L-lysine-forming) | 33 | 1119 |
| *Cgmri1::Tn7* | 0.06 | *CAGL0D04576g* | *MRI1* | MRI1 5'-methylthioribose-1-phosphate isomerase | 959 | 1248 |
| **Chromosome segregation** | | | | | | |
| *Cgnud1::Tn7* | 0.07 | *CAGL0D06644g* | *NUD1* | Component of the spindle pole body outer plaque | 54 | 2703 |
| *Cgsds22::Tn7* | 0.10 | *CAGL0D00264g* | *SDS22* | Nuclear regulatory subunit of Glc7p type 1 protein serine-threonine phosphatase | 1089 | 1212 |
| **Ion transport** | | | | | | |
| *Cgena1::Tn7* | 0.02 | *CAGL0K12034g* | *ENA1* | P-type ATPase sodium pump, involved in Na+ and Li+ efflux | 1185 | 3264 |
| *Cggtr1::Tn7* | 0.10 | *CAGL0B02453g* | *GTR1* | Cytoplasmic GTP binding protein and negative regulator of the Ran/Tc4 GTPase cycle | 635 | 951 |
| **Protein targeting** | | | | | | |
| *Cghsp78::Tn7* | 0.03 | *CAGL0M08822g* | *HSP78* | Oligomeric mitochondrial matrix chaperone | 2245 | 2364 |
| *Cglhs1::Tn7* | 0.06 | *CAGL0F06369g* | *LHS1* | Molecular chaperone of the endoplasmic reticulum lumen | 2240 | 2670 |
| **Carbohydrate metabolic process** | | | | | | |
| *Cghxk2::Tn7* | 0.03 | *CAGL0A04829g* | *HXK2* | Hexokinase isoenzyme 2 | 382 | 1461 |
| **Cellular ion homeostasis** | | | | | | |
| *Cghal5::Tn7* | 0.07 | *CAGL0M08360g* | *HAL5* | Putative protein kinase | 1822 | 2301 |
| **Cofactor metabolic process** | | | | | | |
| *Cgcab5::Tn7* | 0.09 | *CAGL0F05555g* | *CAB5* | Putative dephospho-CoA kinase | 646 | 720 |
| **DNA replication** | | | | | | |
| *Cgcti6::Tn7* | 0.09 | *CAGL0C01485g* | *CTI6* | Protein that relieves transcriptional repression by binding to the Cyc8-Tup1 corepressor and recruiting the SAGA complex to the repressed promoter | 71 bps from start codon | 1623 |
| **Endosome transport** | | | | | | |
| *Cgrrt2::Tn7* | 0.02 | *CAGL0C01661g* | *RRT2* | Putative protein of unknown function | 319 | 1128 |
| **Lipid metabolic process** | | | | | | |
| *Cginp53::Tn7* | 0.03 | *CAGL0B04631g* | *INP53* | Polyphosphatidylinositol phosphatase | 2894 | 3291 |
| **Nuclear transport** | | | | | | |
| *Cgsxm1::Tn7* | 0.08 | *CAGL0M01496g* | *SXM1* | Nuclear transport factor (karyopherin) | 2397 | 2856 |
| **Regulation of cell cycle** | | | | | | |
| *Cgpin4::Tn7* | 0.08 | *CAGL0I05082g* | *PIN4* | Protein involved in G2/M phase progression | 197 | 1815 |
| **RNA catabolic process** | | | | | | |
| *Cgski1::Tn7* | 0.01 | *CAGL0K05511g* | *SKI7* | Putative GTPase | 1091 | 2184 |
| **RNA modification** | | | | | | |
| *Cggcd14::Tn7* | 0.05 | *CAGL0L05566g* | *GCD14* | Subunit of tRNA (1- methyl adenosine) methyltransferase | 668 | 1125 |
| **Transcription from RNA polymerase II promoter** | | | | | | |
| *Cgnut1::Tn7* | 0.07 | *CAGL0E05060g* | *NUT1* | Component of the RNA polymerase II mediator complex | 487 | 3300 |
| **Miscellaneous processes** | | | | | | |
| *cagl0h00682g::Tn7* | 0.06 | *CAGL0H00682g* | *YMR196w* | Putative protein of unknown function | 1183 | 3141 |
| *cagl0i07535g::Tn7* | 0.10 | *CAGL0I07535g* | YOL098c | Putative metalloprotease | 3066 | 1700 |
| *cagl0l10852g::Tn7* | 0.09 | *CAGL0L10582g* | *YMR196w* | Putative protein of unknown function | 3135 | 3297 |
| *Cgapl3::Tn7* | 0.02 | *CAGL0F04389g* | *APL3* | Alpha-adaptin, large subunit of the clathrin associated protein complex | 1723 | 3022 |
| *Cgkcs1::Tn7* | 0.00 | *CAGL0D04378g* | *KCS1* | Inositol hexakisphosphate and inositol heptakisphosphate kinase | 371 | 3162 |
| *Cgpdr12::Tn7* | 0.03 | *CAGL0M07293g* | *PDR12* | Plasma membrane ATP-binding cassette transporter | 2274 | 4548 |
| *Cgsef1::Tn7* | 0.08 | *CAGL0A04455g* | *SEF1* | Putative transcription factor | 254 | 3291 |
