# Supplementary material for: Functional Genomic Analysis of Candida glabrata-Macrophage Interaction: Role of Chromatin Remodeling in Virulence
Source: PLoS Pathog. 2012 Aug 16;8(8):e1002863. doi: 10.1371/journal.ppat.1002863 (PMC3420920; doi:10.1371/journal.ppat.1002863)
Supplement: Table S3 — List of strains and plasmids used in the study. (DOCX) [file ppat.1002863.s013.docx]

**Table S3: List of strains and plasmids used in the study**

| **Strain** | **Genotype** | **Reference** |
| --- | --- | --- |
| **Yeast** | | |
| YRK19 | *ura3∆::Tn903* G418^R^ | 3 |
| YRK20 | *URA3* | 4 |
| YRK103 | *ura3∆::Tn903 G418^R^Cgyps1-11∆::hph* | 5 |
| YRK220 | *ura3∆::*Tn903 G418^R^/pBRK202 | This study |
| YRK402 | *ura3∆::*Tn903 G418^R^*Cgrtt107Δ::hph* | This study |
| YRK422 | *URA3 Cgrsc-aΔ::hph* | This study |
| YRK646 | *ura3∆::*Tn903 G418R *Cgrtt107Δ::hph*/pBRK700 | This study |
| YRK649 | *ura3∆::Tn903 G418^R^ Cgyps1-11∆::hph/*pBRK202 | This study |
| YRK659 | *ura3∆::*Tn903 G418^R^ *Cgrtt109∆::nat1* | This study |
| YRK668 | *URA3 Cgrsc3-aΔ::hph Cgrsc3-bΔ::nat1* | This study |
| YRK691 | ura3∆::Tn903 G418^R^ *Cgrsc3-bΔ::nat1* | This study |
| YRK693 | *URA3 Cgsgs1∆::nat1* | This study |
| YRK713 | *ura3∆::*Tn903 G418R *Cgacs1Δ::nat1* | This study |
| YRK753 | *ura3∆::*Tn903 G418R *Cgrtt107Δ::hph*/pRK202 | This study |
|  |  |  |
| **Tn*7* Transposon** | | |
| Tn*7* Transposon | Tn*7* R6Kγ ori *URA3 npt* (Km^R^) | 1 |
| YRK588 | *Cgchz1::*Tn*7* | This study |
| YRK589 | *Cgarp7::*Tn*7* | This study |
| YRK590 | *Cgcti6::*Tn*7* | This study |
| YRK591 | *Cggtr1::*Tn*7* | This study |
| YRK593 | *Cgrsc3-b::*Tn*7* | This study |
| YRK594 | *Cgdna2::*Tn*7* | This study |
| YRK596 | *Cgrsc3-a::*Tn*7* | This study |
| YRK597 | *Cgsgs1::*Tn*7* | This study |
| YRK608 | *Cghfi1::*Tn*7* | This study |
| YRK755 | *Cgrtt107::*Tn*7* | This study |
| YRK547 | *Cgrtt109::*Tn*7* | This study |
|  |  |  |
| **Bacteria** |  |  |
| BW23473 | *∆lac-169 robA1creC510hsd*R514 uidA::*pir endA recA* | 6 |
| **Plasmid** | **Description** | **Reference** |
| pRK74 | A CEN-ARS plasmid (pGRB2.2) of *C. glabrata* carrying *S.cerevisiaeURA3* as a selection marker. MCS sites are flanked by *S. cerevisiaePGK1* promoter at one end and by 3' UTR of HIS3 at the other end. | 7 |
| pRK202 | GFP expressing *C. glabrata* plasmid | Cormack laboratory |
| pAP599 | Contains *URA3* marker and an *hph* expression cassette (The *hph* gene is present between *S. cerevisiae PGK1* promoter and *HIS3* 3' UTR). The *hph* cassette confers hygromycin resistance (Hyg^R^). | 8 |
| pRK588 | 5' UTR and 3' UTR of *CgRTT107* cloned in pAP599 | This study |
| pRK613 | 5' UTR and 3’ UTR of *CgRSC3-A* (*CAGL0D03850g*) cloned in pAP599 | This study |
| pRK849 | *CgRSC3-A* ORF (2.5 kb) cloned in *SpeI-BamHI* sites in pRK74 | This study |
| pRK806 | *CgRSC3-B* ORF (2.4 kb) cloned in *XmaI-XhoI* sites in pRK74 | This study |
| pRK700 | *CgRTT107* ORF (3.3 kb) cloned in *SmaI-SalI* sites in pRK74 | This study |
| pRK941 | *CgRTT109* ORF cloned (1.3 kb) in *BamHI-SalI* sites in pRK74 | This study |
