# Supplementary material for: Functional Genomic Analysis of Candida glabrata-Macrophage Interaction: Role of Chromatin Remodeling in Virulence
Source: PLoS Pathog. 2012 Aug 16;8(8):e1002863. doi: 10.1371/journal.ppat.1002863 (PMC3420920; doi:10.1371/journal.ppat.1002863)
Supplement: Table S4 — List of antibodies used in the study. (DOCX) [file ppat.1002863.s014.docx]

**Table S4: List of antibodies used in the study**

| **Name** | **Dilution used** | **Clonality** | **Company** | **Catalog number** |
| --- | --- | --- | --- | --- |
| **Primary antibodies** | | | | |
| Anti-gapdh | 1:10,000 | Polyclonal | Abcam | ab22555 |
| Anti-histone 1 | 1:1000 | Monoclonal | Abcam | ab62884 |
| Anti-histone H2A | 1:2000 | Polyclonal | Abcam | ab18255 |
| Anti-histone H2B | 1:5000 | Polyclonal | Abcam | ab1790 |
| Anti-histone H3 | 1:10,000 | Polyclonal | Millipore | 06-755 |
| Anti-histone H4 | 1:500 | Polyclonal | Abcam | ab10158 |
| Anti-H3K9Ac | 1:1000 | Polyclonal | Cell Signaling Technology | 9649 |
| Anti-H3K14Ac | 1:5000 | Polyclonal | Millipore | 06-911 |
| Anti-H3K56Ac | 1:1000 | Monoclonal | Abcam | ab76307 |
| Anti-H3K9Me_3_ | 1:5000 | Polyclonal | Cell Signaling Technology | 9754 |
| Anti-H3K27Me_2_ | 1:5000 | Monoclonal | Cell Signaling Technology | 9728 |
| Anti-H4K16Ac | 1:500 | Polyclonal | Abcam | ab61240 |
| Anti-H4K20Me_3_ | 1:10,000 | Polyclonal | Abcam | ab9053 |
| Anti-H3S10P | 1:1000 | Polyclonal | Cell Signaling Technology | 9701S |
| Anti-gamma H2AX | 1:5000 | Monoclonal | Abcam | ab11174 |
| Anti-acetylated lysine | 1:500 | Polyclonal | Abcam | ab23364 |
| **Secondary antibodies** | | | | |
| Anti-mouse | 1:5000 |  | Cell Signaling Technology | 7074S |
| Anti-rabbit | 1:5000 |  | Cell Signaling Technology | 7076S |
