# Supplementary material for: Functional Genomic Analysis of Candida glabrata-Macrophage Interaction: Role of Chromatin Remodeling in Virulence
Source: PLoS Pathog. 2012 Aug 16;8(8):e1002863. doi: 10.1371/journal.ppat.1002863 (PMC3420920; doi:10.1371/journal.ppat.1002863)
Supplement: Table S5 — Primers used in the study. (DOCX) [file ppat.1002863.s015.docx]

**Table S5: List of primers used in the study**

| **Primer name** | **Sequence (5’-3’)** | **Target gene** |
| --- | --- | --- |
| **For creating knockouts and confirmation by PCR** | | |
| OgRK 308 | CGCTCGAGCGATCTATCTGCTACTCTAGCAGAG | *CgRSC3-A* |
| OgRK 310 | ATAAGAATGCGGCCGCTCTTTTCAATTCTGGTGTTATGGA | *CgRSC3-A* |
| OgRK 311 | CGGAGCTCCGATTGATATGCTCTTGCAGTTT | *CgRSC3-A* |
| OgRK 312 | CTGGGAGCGTTCAGATCTTC | *CgRSC3-A* |
| OgRK 313 | CACCCGCATTGATTGTAATG | *CgRSC3-A* |
| OgRK 314 | AGGTGCGAACGTCTACCAAT | *CgRSC3-A* |
| OgRK 315 | GTAATCGGTCCCGCACTGC | *CgRSC3-A* |
| OgRK 309 | CGCAAGCTTCGTATTATACCGGATCAAAT | *CgRSC3-A* |
| OgRK 621 | CAATTCATGATTCAAGGTGGTG | *CgRSC3-B* |
| OgRK 622 | ACCACTGTTGCCACACCAT | *CgRSC3-B* |
| OgRK 623 | GCGTCGACCTGCAGCGTACGTGCCTCTAACTTGTATAGATCAA | *CgRSC3-B* |
| OgRK 624 | CGACGGTGTCGGTCTCGTAGCCTTTTAATGAAGCAAGGGACT | *CgRSC3-B* |
| OgRK 625 | TGGGAAACTTGGCTACTTGC | *CgRSC3-B* |
| OgRK 626 | AAACAAGGATGCCCGTCA | *CgRSC3-B* |
| OgRK 591 | ATGATCTGTTCAATTTCAATAC | *CgRSC3-B* |
| OgRK 592 | CCATAGAGCAGTGTATCTTAA | *CgRSC3-B* |
| OgRK 299 | CGCTCGAGGTGCAGTAGTGAACTTATCGCAAGCAA | *CgRTT107* |
| OgRK 300 | CGCAAGCTTTTCATTTACTACCAGTTTGATAATTCAA | *CgRTT107* |
| OgRK 301 | GCCGCCGGCGGCCGCGGCATGCAAGGCATGTTA | *CgRTT107* |
| OgRK 302 | CGGAGCTCGTGCAGCCTCGATGCTCTATTTACAAG | *CgRTT107* |
| OgRK 303 | GTATCGAAAGCCACATTATTGA | *CgRTT107* |
| OgRK 304 | GTTCAACATGGGATTGCATTG | *CgRTT107* |
| OgRK 305 | ACAATCTAGGGCCCATGACC | *CgRTT107* |
| OgRK 306 | AGCTTTCGCTTTCCCTGATA | *CgRTT107* |
| OgRK 579 | GCAATTGAGGCCGATAAAAC | *CgRTT109* |
| OgRK 580 | GGTAAACCAACTAAACATCATACTGG | *CgRTT109* |
| OgRK 581 | GCGTCGACCTGCAGCGTACGCGTATTTTGATTGGACTTTCTGA | *CgRTT109* |
| OgRK 582 | CGACGGTGTCGGTCTCGTAGTTCTGAGAACTAAAGAAAAACTTCAA | *CgRTT109* |
| OgRK 583 | AGGGCTCTCAACTTTTTCTGA | *CgRTT109* |
| OgRK 584 | TCGTTCAAGGATGTTGTGGA | *CgRTT109* |
| OgRK 585 | AGCGGATACCAATGGATACTG | *CgRTT109* |
| OgRK 586 | GGAAGCCCACCATTTCAATA | *CgRTT109* |
| OgRK 573 | TTCAAAATCAGCCAGTATGAGC | *CgSGS1* |
| OgRK 574 | AGGGCGAAGTTGACACATCT | *CgSGS1* |
| OgRK 575 | GCGTCGACCTGCAGCGTACGTCTAAACAAGTGATTAGGCC | *CgSGS1* |
| OgRK 576 | CGACGGTGTCGGTCTCGTAGTTTTTATCCATTATTTAGTTTGGACA | *CgSGS1* |
| OgRK 577 | GTGCTTCCATGGAGGTTGAT | *CgSGS1* |
| OgRK 578 | GGTGGTGACGGTAGTGTGC | *CgSGS1* |
| OgRK 607 | GGAATCCTTCGAAGTGTATCTT | *CgSGS1* |
| OgRK 608 | TCTCTTTAATAGGATCAGGGAGTT | *CgSGS1* |
| OgRK 340 | CGTACGCTGCAGGTCGACGCCTTCCGCTGCTAGGCGCGCCGTG | *nat1* |
| OgRK 341 | CTACGAGACCGACACCGTCGGGCCGCTGACGAAGT | *nat1* |
| OgRK 342 | GTCTACTACTTTGGATGATAC | *nat1* |
| OgRK 343 | TCTGTTCCAACCAGAATAAG | *nat1* |
| **For qRT-PCR** | | |
| OgRK 420 | GTCGTCTTCGCAGGTTTCTC | *CgACS1* |
| OgRK 421 | TTGTGGGGTTTCTCTCAAGG | *CgACS1* |
| OgRK 839 | AGACCGCTGTGGTTGGTATC | *CgACS2* |
| OgRK 840 | AGCGAATGGACCAATTTCAC | *CgACS2* |
| OgRK 686 | CTACTAAAGCGTGTTGGTGACG | *CgARN1* |
| OgRK 687 | GGCAACAATTGTCTCCAGTCTT | *CgARN1* |
| OgRK 944 | TTGGGAAGGTGACAACAACA | *CgCMK1* |
| OgRK 945 | AGGTGAACACTTTCCGGATG | *CgCMK1* |
| OgRK 680 | TTAGGTTTGATGATGCACGAAG | *CgFTR1* |
| OgRK 681 | TGTTGTCCATACGGTTTAGCAG | *CgFTR1* |
| OgRK 392 | AGCTGGTTCAGCTGCTAAGG | *CgHTA1* |
| OgRK 393 | TTGCAAATGTCTTGGGATGA | *CgHTA1* |
| OgRK 394 | GGTTCTGCTGCTAAGGCTTC | *CgHTA2* |
| OgRK 395 | GCCAATTCCAAGATTTCAGC | *CgHTA2* |
| OgRK 455 | GAAGAGAACCAAGGCCAGAA | *CgHTB1* |
| OgRK 456 | GCGGTTTGGATTTCTCTAGC | *CgHTB1* |
| OgRK 396 | TGCTAAAGCCGAAAAGAAGC | *CgHTB2* |
| OgRK 397 | GGTGTCTGGGTGAGTTTGCT | *CgHTB2* |
| OgRK 402 | GATTCCAATCCTCTGCCATC | *CgHHT1* |
| OgRK 403 | CACCTCTCAATCTTCTAGCCAAC | *CgHHT1* |
| OgRK 404 | AGCCAGGTACTGTCGCTTTG | *CgHHT3* |
| OgRK 405 | ATGGCAGCCAAGTTGGTATC | *CgHHT3* |
| OgRK 459 | GAATCTGTTATCAGAGACGCTGT | *CgHHF2* |
| OgRK 460 | TTAACCACCGAAACCGTACA | *CgHHF2* |
| OgRK 408 | TCAAGGTATTACCAAGCCAGCTA | *CgHHF3* |
| OgRK 409 | TTCTCTTGGCGTGTTCAGTG | *CgHHF3* |
| OgRK 416 | CTTCTTCGACTGGGACTTGC | *CgICL1* |
| OgRK 417 | GTCTGGGTACTTGGCCTTGA | *CgICL1* |
| OgRK 940 | CACCTAAGGTCGCTGTGTCA | *CgISA1* |
| OgRK 941 | TTCTCCACCCAGTCCATCTC | *CgISA1* |
| OgRK 418 | CATCACTTGGAGGCCAAACT | *CgMLS1* |
| OgRK 419 | AGTCCCAACGACCACAGTTC | *CgMLS1* |
| OgRK 942 | TTGGGAAGGTGACAACAACA | *CgPOX1* |
| OgRK 943 | AGGTGAACACTTTCCGGATG | *CgPOX1* |
| OgRK 930 | GCAAAAATGGATCCCTGAGA | *CgREX2* |
| OgRK 931 | GATTGTGTCTCCTGGCCACT | *CgREX2* |
| OgRK 65 | CGACGACCCATCCCCAGGCTC | *CgYPS3* |
| OgRK 66 | ACTTAGCTCTTCATGGTAACG | *CgYPS3* |
| **Cloning primers** | | |
| OgRK 465 | GCACTAGTTGATCCGGTATAATACGCAATGTACC | *CgRSC3-A* |
| OgRK 466 | GCACTAGTTGATCCGGTATAATACGCAATGTACC | *CgRSC3-A* |
| OgRK 639 | CGCCCGGGCAATGGATATTCGTGGTCGCAAA | *CgRSC3-B* |
| OgRK 640 | CGCTCGAGCATTAAAAGGATATCAAGAACA | *CgRSC3-B* |
| OgRK 360 | AGTAAACCCGGGATGTCTATTGATACATCGAT | *CgRTT107* |
| OgRK 361 | ACTCCGTCGACTGTACTAAATCCTTTTATTGA | *CgRTT107* |
| OgRK 633 | CAGGATCCATGCTCCAAGCAATTCTCAAAG | *CgRTT109* |
| OgRK 634 | GGGTCGACCTATTTCTTTCTGCGAACTTGGA | *CgRTT109* |
