# Supplementary material for: Functional Genomic Analysis of Candida glabrata-Macrophage Interaction: Role of Chromatin Remodeling in Virulence
Source: PLoS Pathog. 2012 Aug 16;8(8):e1002863. doi: 10.1371/journal.ppat.1002863 (PMC3420920; doi:10.1371/journal.ppat.1002863)
Supplement: Text S1 — Materials and Methods and References for Supplementary Information. (DOC) [file ppat.1002863.s016.doc]

**Supporting Information**

1. Supplemental methods
2. Supplemental references
3. Supplementary figures: S1 to S10

**1. Supplemental methods**

**Strains and culture conditions**

Bacterial and yeast strains were routinely maintained in LB medium at 37°C and in YPD medium at 30°C, respectively unless otherwise stated.

**ROS measurement**

*C. glabrata* infected THP-1 cells were incubated with 50 µM CM-H2DCFDA (5-(and 6-)-chloromethyl-2,7-dichlorodi- hydroﬂuorescein diacetate, acetyl ester) for 1 h at 37°C, washed and lysed. Lysates were centrifuged at 4000 rpm for 5 min, quantified for the protein content and fluorescence of the oxidized product dichloroﬂuorescein was measured on Varioskan with excitation and emission at 490 nm and 530 nm, respectively. For data representation, ﬂuorescence units were normalized to the total protein amount.

**Fluorescence microscopy**

PMA-treated THP-1 cells were infected with GFP expressing *C. glabrata* at 1:2 MOI for 1 h, PBS-washed, and incubated with 100 nM LysoTracker Red (DND-99) for 30 min. Cells are fixed with 3.7% formaldehyde for 20 min before visualization. For DAPI (49,6-diamidino-2-phenylindole) staining, fixed cells were permeabilized with 0.7% Triton X-100 for 15 min and imaged in Vectashield mounting medium containing DAPI. Heat-killed yeast cells were prepared by incubating *C. glabrata* cell suspensions at 95°C for 10 min. The slides were examined with a laser scanning microscope (LSM 510 Meta; Carl ZEISS).

**Membrane preparation for the STM screen**

Plasmid DNA was isolated from 96 *Escherichia coli* strains each harbouring a plasmid containing the unique 60-mer oligonucleotide (tag) sequence and 200 ng of alkali-denatured plasmid DNA was spotted on Hybond-Nylon membrane using 96-well Dot Blot apparatus. Membranes were neutralised in 2X SSC followed by UV cross-linking of the spotted plasmid DNA. Quality controls were performed to test an equal loading of each plasmid (signature tag sequence) on the membrane and to rule out any cross-hybridization between different tag sequences. P^32^-labelled unique signature tags, amplified from the input and the output yeast samples, were hybridized to the membranes and the signal intensity for each spot was quantified. Mutants with an output/input ratio of ≥ 6.0 and ≤ 0.1 were selected as ‘up’ and ‘ down mutants which represent increased survival with ≥ 6-fold higher representation and reduced survival with ≤ 10-fold reduced representation in the output pool compared to the input pool, respectively. Probed membranes were reused for ~ 10 times after stripping the labelled probe by incubating twice in 0.4 M NaOH at 45°C for 30 min. Mapping and integration of Tn*7* insertion in the mutant genomes *via* homologous recombination was confirmed by PCR as described previously (1). As an additional control for the STM screen, five mutants were regenerated in the *wt* background and their survival profiles in macrophages were found to be similar to those of the respective parental mutant strains (data not shown).

**Phenotypic profiling**

Phenotypic characterization of identified mutants was conducted individually in 96-well plate format. Mutant cultures were grown in YPD medium for overnight, diluted 150-fold in PBS and 5 µl of cell suspension was spotted on different plates with a 96-pin replicator. Growth was recorded after 1-2 days of incubation at 30°C.

**Quantitative Real-time PCR (qRT-PCR)**

Primers for real time PCR analysis were designed using Primer3 plus software and are listed in table S5. To isolate RNA from macrophage-ingested *C. glabrata*, the infected THP-1 cells were washed twice with PBS and lysed in 1 ml ice-cold water. The lysate was centrifuged followed by two quick washes with DEPC-treated water. The washed yeast cell pellets were frozen on dry ice and cells were disrupted with glass beads in trizol. Total RNA was isolated using acid phenol extraction method and qRT-PCR was performed as described previously (2). Results were normalized to GAPDH gene signal and comparisons were made between the C_t_ values of RPMI-grown and macrophage-internalized samples and fold difference in expression was calculated. Since *CgICL1* is known to be induced upon internalization by murine macrophages, its transcript levels were examined as a control (Figure 2C). Notably, cDNA prepared from internalized yeast yielded no amplification for the human genes coding for Ccl5 and histone H3, thus, eliminating the possible contamination of THP-1 RNA with yeast RNA.

**Mouse infection assay**

Experiments involving mice were conducted at VIMTA Labs, Hyderabad. 100 μl of YPD-grown *C.* *glabrata* cells (4 X 10^7^) was injected into female BALB/c mice (6-8 weeks old) through tail vein. Seven days post infection, mice were sacrificed and kidneys, liver and spleen were harvested. The organs were homogenized in 1 ml PBS and fungal burden was assessed by plating tissue homogenate on YPD plates containing penicillin and streptomycin. All mice experiments were repeated twice with a set of 7-8 mice per strain in each experiment.

**Cloning of *C. glabrata* ORFs**

*CgRSC3-A* (2.5 kb)*, CgRSC3-B* (2.4 kb), *CgRTT107* (3.3 kb) and *CgRTT109* (1.3 kb) ORFs were PCR amplified from genomic DNA of the wild type strain using high fidelity Platinum Pfx DNA polymerase with primers carrying restriction sites for SpeI-BamHI, XmaI-XhoI, SmaI-SalI and BamHI-SalI for *CgRSC3-A*, *CgRSC3-*B, *CgRTT107* and *CgRTT109*, respectively. Amplified fragments were cloned, downstream of the *PGK1* promoter, in the pGRB2.2 plasmid. Clones were verified by PCR, sequencing and complementation analysis.

**Other procedures**

*C. glabrata* transformation, growth and viability assays, ORF disruption by homologous recombination were performed as described previously (2).

**2. Supplemental references**

1. Castaño I, Kaur R, Pan S, Cregg R, De Las Peñas A. et al. (2003) Tn*7*-based genome-wide random insertional mutagenesis of *Candida* *glabrata*. Genome Res 13: 905–915.
2. Borah S, Shivarathri R, Kaur R (2011) The Rho1 GTPase-activating protein CgBem2 is required for survival of azole stress in *Candida glabrata*. J. Biol Chem 286: 34311-34324.
3. Cormack BP, Falkow S (1999). Efficient homologous and illegitimate recombination in the opportunistic yeast pathogen *Candida glabrata*. Genetics 151: 979-987.
4. De Las Penas, A, Pan, SJ, Castano I, Alder J, Cregg R, et al. (2003). Virulence-related surface glycoproteins in the yeast pathogen *Candida glabrata* are encoded in subtelomeric clusters and subject to RAP1- and SIR-dependent transcriptional silencing. Genes Dev 17: 2245–2258.
5. Kaur R, Ma B, Cormack, BP (2007). A family of glycosylphosphatidylinositol-linked aspartyl proteases is required for virulence of *Candida glabrata.*  Proc Natl Acad Sci USA 104: 7628-7633.
6. Metcalf WW, Jiang W, Daniels LL, Kim SK, Haldimann A, et al. (1996). Conditionally replicative and conjugative plasmids carrying lacZ alpha for cloning, mutagenesis, and allele replacement in bacteria*.* Plasmid 35: 1–13.
7. Frieman MB, McCaffery JM, Cormack, BP (2002). Modular domain structure in the *Candida glabrata* adhesin Epa1p, a beta1, 6 glucan-cross-linked cell wall protein. Mol Microbiol 46: 479–492.
8. Ma B, Pan S, Domergue R, Rigby T, Whiteway, M, et al. (2009). High-affinity transporters for NAD^+^ precursors in *Candida glabrata* are regulated by Hst1 and induced in response to niacin limitation. Mol Cell Biol 29: 4067-4079.

**3. Supplementary figures S1 to S10**
